# Novel Steroidal 5α,8α-Endoperoxide Derivatives with Semicarbazone/Thiosemicarbazone Side-chain as Apoptotic Inducers through an Intrinsic Apoptosis Pathway: Design, Synthesis and Biological Studies

**DOI:** 10.3390/molecules25051209

**Published:** 2020-03-07

**Authors:** Liwei Ma, Haijun Wang, Jing Wang, Lei Liu, Song Zhang, Ming Bu

**Affiliations:** 1 Research Institute of Medicine & Pharmacy, Qiqihar Medical University, Qiqihar 161006, China; maliwei@qmu.edu.cn; 2 College of Pharmacy, Qiqihar Medical University, Qiqihar 161006, China; wanghaijun@qmu.edu.cn (H.W.); qyyx305@qmu.edu.cn (J.W.); liuleiqiyi@sina.com (L.L.); 3 Basic Medical Science College, Qiqihar Medical University, Qiqihar 161006, China; zhangsong@qmu.edu.cn

**Keywords:** ergosterol peroxide, semicarbazone, thiosemicarbazone, anti-tumor activity, apoptosis, HepG2 cells

## Abstract

A series of novel steroidal 5α,8α-endoperoxide derivatives bearing semicarbazone (**7a**–**g**) or thiosemicarbazone (**7h**–**k**) side chain were designed, synthesized and evaluated for their cytotoxicities in four human cancer cell lines (HepG2, HCT-116, MCF-7, and A549) using the MTT assay *in vitro*. The results showed that compound **7j** exhibited significant cytotoxic activity against HepG2 cells (IC_50_ = 3.52 μM), being more potent than ergosterol peroxide. Further cellular mechanism studies in HepG2 cells indicated that compound **7j** triggered the mitochondrial-mediated apoptosis by decreasing mitochondrial membrane potential (MMP), which was associated with up-regulation of Bax, down-regulation of Bcl-2, activation levels of the caspase cascade, and formation of reactive oxygen species (ROS). The above findings indicated that compound **7j** may be used as a promising skeleton for antitumor agents with improved efficacy.

## 1. Introduction

**Figure 1:**
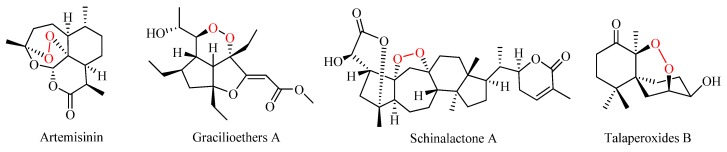
Representative compounds of natural endoperoxides with bioactivities.

Cancer is a serious disease that poses a risk people’s health. The discovery of potent anticancer drugs from natural products is one of the important directions in drug research. Nowadays, it’s still more than 50% of drugs used in the areas of cancer and infectious diseases are based on natural origins [1,2]. Natural endoperoxides are widely found in nature, which all have a distinct peroxide bond (-O-O-) in their structures as cyclic organic compounds, such as artemisinin, schinalactone A, talaperoxides B and gracilioethers A, yingzaosu, *etc*. (Figure 1) [3,4,5]. Most of natural endoperoxides have been proved with antiviral, anticancer or antifungal activity [6,7,8]. The most representative discovery of endoperoxide from natural products is artemisinin by Youyou Tu (2015 Nobel Award winner) [9]. Artemisinin was extracted from a traditional Chinese medicine (*Artemisia annua L*.), and many derivatives of it have been synthesized and used for treatment of malaria [10,11].

Steroidal compounds have drawn attention not only due to their unusual and interesting chemical structures, but also due to their widespread application as anti-inflammatory, diuretic, anabolic, contraceptive, and anticancer agents [12]. Most steroidal drugs in use today are semisynthetic compounds widely used in traditional medicines by modification of the steroid ring system and side chains [13]. The interesting structural and stereochemical features of the steroid nucleus provide additional fascination for researchers, and the introduction of heteroatoms, heterocycles, or amides, or replacement of one or more carbon atoms in the steroidal skeleton has been envisaged to discover new chemical entities with the potential to become promising future drugs, resulting in notable modifications in biological activity [14,15,16].

**Figure 2:**
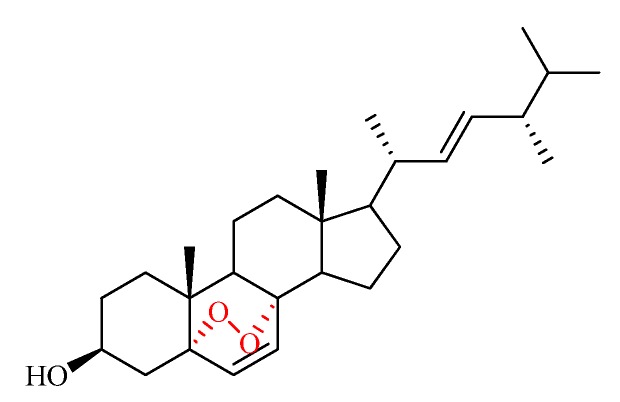
Structure of ergosterol peroxide (EP).

Ergosterol peroxide (5α,8α-epidioxiergosta-6,22-dien-3β-ol, EP) is a member of a class of fungal secondary metaboliters of sterol 5α,8α-endoperoxidederivatives (Figure 2). A number of biological activities have been attributed to EP, including antitumor, anti-inflammatory, antiviral and immunomodulatory activities and antioxidant activities [16,17,18,19,20,21]. As an important active lead compound in drug discovery, EP is well known for its 5α,8α-peroxy moiety. During the last years, our research group has been working on the modifications of steroids to obtained more active compounds as potential antitumor agents. In our previous studies, we synthesized a series of steroidal 5α,8α-endoperoxide derivatives with aliphatic side-chain. We found that most of these derivatives exhibited significant anticancer activities compared to ergosterol, which has no peroxidic bridge at C-5 and C-8 positions with barely inhibition activity against all the tested cells. The studies showed that the 5α,8α-peroxygroup is requisite pharmacophore for these derivatives [22]. Then we synthesized several series of steroidal 5α,8α-endoperoxide derivatives with 17-(*O*-carboxymethyl)oxime side-chain and hydrazone hybrid side-chain. The MTT assay indicated that some derivatives possessing the 5α,8α-peroxy group and different side chains displayed distinct cytotoxic activity against some cancer cell lines [23,24,25,26,27]. Based on the above results, we speculate that the difference between side chains at C-17 position and 5α,8α-peroxy group would provide a synergistic effect for the bioactivity.

**Figure 3:**
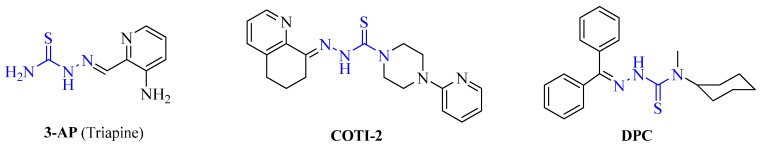
Representative compounds with thiosemicarbazones structure in the clinical trial.

Semicarbazones and thiosemicarbazones are a class of Schiff bases that have been gaining considerable attention due to their wide array of pharmacological effects or biological activities [28,29]. In a literature review, we found that semicarbazone based derivatives have shown anticancer, anti-protozoal, antimicrobial, pesticidal activity, *etc*. [30,31,32,33]. In addition, thiosemicarbazones (TSCs) have a wide clinical antitumor spectrum with efficacy in various tumor types such as bladder cancer, pancreatic cancer, cervical cancer, non-small cell lung cancer, breast cancer, prostate cancer and leukemia [34,35,36,37]. Figure 3 shows some representative compounds with thiosemicarbazones structure in the clinical trial. In addition, it is reported that combining steroids (progesterone and testosterone) with semicarbazide or thiosemicarbazide moieties could be a good anticancer drug [38].

**Figure 4:**
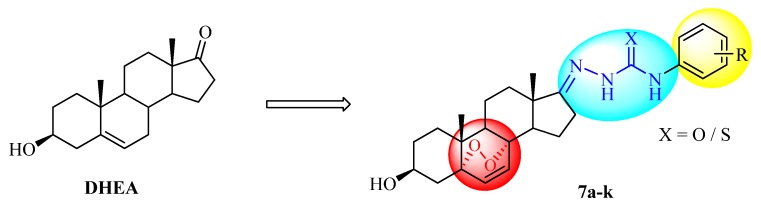
Design strategy of novel steroidal derivatives.

Inspired by the good biological properties of semicarbazones and thiosemicarbazones, and in continuation with our previous work on the modifications of steroids, we here report the design and synthesis of a series of novel steroidal endoperoxide derivatives with semicarbazone/thiosemicarbazone side-chain (Figure 4) and their underlying action mechanisms of anticancer activity, wishing to find new effective antitumor agents.

## 2. Results and Discussion

### 2.1. Chemistry

**Scheme 1:**
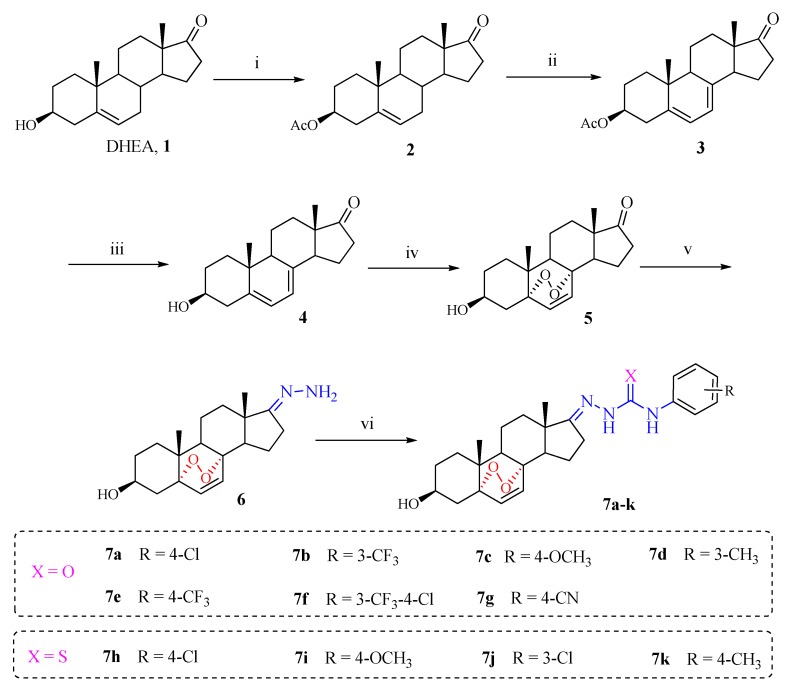
Synthesis of compounds **7a**–**k**. Reagents and conditions: (i) Ac_2_O, pyridine, DCM, rt, 97%; (ii) Cyclohexane, NBS, reflux, 1 h; 2,4,6-collidine, xylene, reflux, 3 h, 33%; (iii) KOH, MeOH, petroleum ether, reflux, 1 h, 93%; (iv) MeOH, O_2_, phloxine B, light, 0°C, 0.5 h, 44%; (v) NH_2_NH_2_-H_2_O, EtOH, 45°C,1 h, 82%; (vi) EtOH, AcOH, different isocyanates and thioisocyanates for **7h**–**k**, rt, 2~5 h, 85%~93%.

The general procedure for the synthesis of steroidal derivatives is shown in Scheme 1. Dehydroepiandrosterone (DHEA, **1**) was reacted with acetic anhydride in the presence of pyridine and dichloromethane to give compound **2**. Compound **2** further underwent bromination with N-Bromosuccinimide (NBS) and debromination with 2,4,6-collidineto afford compound **3**. Compound **3** was reacted with potassium hydroxide for deacetylation to give compound **4**. Subsequently, compound **4** was reacted with oxygen to give endoperoxide **5**, using phloxine B as photosensitizer. Then, compound **5** was reacted with hydrazine hydrate to obtain the corresponding steroidal hydrazine **6**. At last, the resulting hydrazine **6** was reacted with different phenyl isocyanate or isothiocyanate substituents to obtain target novel 5α,8α-endoperoxide steroidal conjugation semicarbazone/thiosemicarbazone derivatives **7a**–**k**. The structures of all compounds were characterized by MS, ^1^H-NMR and ^13^C-NMR spectrum.

### 2.2. Biological Evaluation

#### 2.2.1. *In Vitro* Cytotoxic Activity

All of the newly synthesized steroidal endoperoxide derivatives **7a**–**k** were investigated for their anti-proliferative activities against human hepatocellular carcinomacells (HepG2), human breast carcinoma cells (MCF-7), human colorectal cells (HCT-116), and human lung carcinoma cells (A549) by the MTT assay *in vitro*. Mitomycin was used as positive reference drug. The results of the MTT assay were summarized as IC_50_ values in Table 1.

The results showed that most of the synthesized derivatives displayed significant cytotoxic activities against all the four tested tumor cell lines, and were more potent than ergosterol peroxide. In addition, it seems that most compounds exhibited significant anti-proliferative activities against HepG2 and MCF-7 cell lines than HCT-116 and A549 cell lines. The results suggested the following rough structure-activity relationships considerations.

For HepG2 cell line, compounds **7j**, **7f**, **7h**, **7b**, **7g**, **7e** and **7a** were the most active compounds with IC_50_ values from 3.52 to 8.65 μM. Compounds **7d**, **7i** and **7k** displayed moderate inhibitory effects with IC_50_ values ranged from 14.35 to 37.50 μM. Compounds **7j** and **7f** were 6.1- and 4.9-fold more potent than ergosterol peroxide, respectively. In addition, compounds **7a**, **7b**, **7e**, **7f**, **7g**, **7h** and **7j** with 4-chlorophenyl, 3-(trifluoromethyl)phenyl, 4-(trifluoromethyl)phenyl, (4-chloro-3-trifluoromethyl)phenyl, 4-cyanophenyl and 3-chlorophenyl substituents were more potent than compounds **7c**, **7d**, **7i** and **7k** with 4-methoxyphenyl and *p*-tolyl, which probably means the electron withdrawing nature of the substituents present on above compounds contributes additional effects on cancer cell growth inhibition.

For MCF-7 cell lines, compounds **7j**, **7f**, **7h**, **7b** and **7g** were the most active compounds with IC_50_ values from 4.88 to 8.98 μM. Compounds **7a** (IC_50_ = 19.31 μM) and **7e** (IC_50_ = 14.90 μM) showed similar cytotoxicity to ergosterol peroxide. However, the activities of compounds **7c**, **7d**, **7i** and **7k** were reduced. For A549 cell line, **7f**, **7j** and **7h** also exhibited significant cytotoxic activities lower than 10 μM. A similar situation was found in A549, HepG2, MCF-7 and HCT-116 cell lines, where the activities of most of compounds **7c**, **7d**, **7i** and **7k** with electron donating groups were markedly reduced (IC_50_> 60 μM).

Taken together, it is notable that compounds **7b** and **7f** with semicarbazone side-chain, **7h** and **7j** with thiosemicarbazone side-chain exhibited more obvious cytotoxic activity to all tested cells as compared to ergosterol peroxide, which suggests that both conjugated N,N,S-tridentate donor set and N,N,O-tridentate donor set are essential for the biological activities of these novel derivatives. In addition, the activity of the compounds was greatly influenced by the change in substitutions at different positions of phenyl ring.

Among the compounds under study, the semicarbazonederivative (**7f**) and thiosemicarbazone derivative (**7j**) were the most potent compounds against HepG2 cell lines (IC_50_< 5 μM). To further investigate the cellular mechanism of these new compounds, compound **7j** was chosen for subsequent biological functions experiments in HepG2 cells.

#### 2.2.2. Compound **7j** Induces Apoptosis in HepG2 Cells

**Figure 5:**
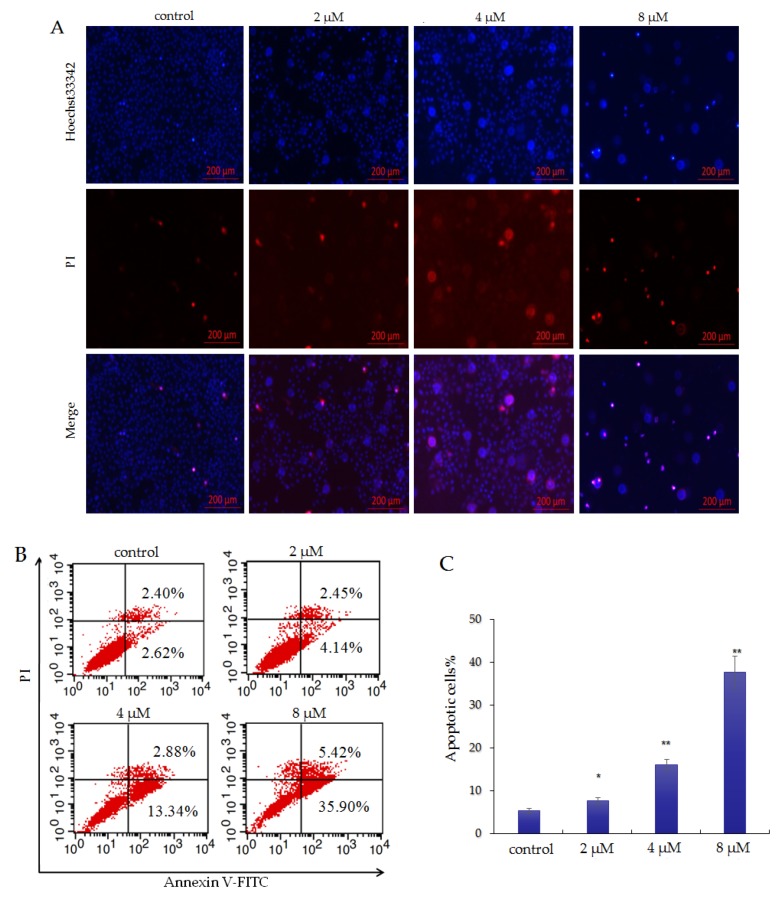
Compound **7j** induced HepG2 cells apoptosis. (**A**) HepG2 cells were treated with compound **7j** (2, 4 and 8 μM) for 48 h and then stained with Hoechst 33342/PI to reflect changes in cell morphology (Scale bar: 200 μm). (**B**) Apoptotic HepG2 cells were detected with Annexin V-FITC/PI staining. Quantitative analysis of apoptosis rates by flow cytometry. (**C**) The bar graph represents the apoptotic cell population at various concentrations. Data were expressed as mean ± SEM, ^*^p< 0.05, ^**^p< 0.01 versus control. The control group was dimethyl sulfoxide (DMSO) solution with 0 μM of **7j**.

Apoptosis is a major type of cell death. In our previous studies, we have reported that ergosterol peroxide exhibited cytotoxic activity against HepG2 and MCF-7 cells by inducing apoptosis [20,21]. In this work, the changes of the morphological characters in HepG2 cells were studies by Hoechst33342/PI staining, which to prove that the inhibitory activity of compound **7j** was related to the inducing of cell apoptosis. As shown in Figure 5A, staining the cells with Hoechst 33342 showed the typical features of apoptosis such as chromatin condensation, and formation of apoptotic bodies. To further demonstrate whether **7j** could induce apoptosis in HepG2 cells, **7j**-treated HepG2 cells were doubly stained with Annexin V-FITC and propidium iodide (PI). As shown in Figure 5B, the percentage of total apoptotic and necrotic cells increased to 16.22% and 41.32% in a dose-dependent manner after treatment with **7j** at the concentrations of 4 and 8 μM, respectively. The results suggested that **7j** exerted its inhibitory effects on proliferation by inducing HepG2 cells apoptosis.

#### 2.2.3. Compound **7j** Induces Mitochondrial Membrane Potential (MMP) Loss in HepG2 Cells

**Figure 6:**
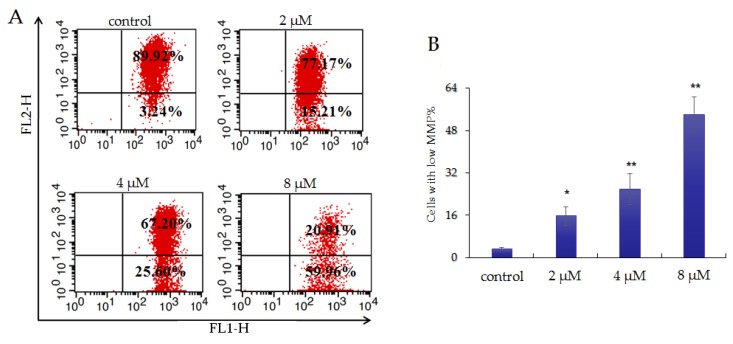
Compound **7j** induced mitochondrial depolarization of HepG2 cells. (**A**) The HepG2 cells were treated with **7j** (2, 4 and 8 μM) for 24 h followed by incubation with the fluorescence probe JC-1 for 30 min. Then, the cells were detected by flow cytometry. (**B**) The bar graph represents the low MMP cell population at various concentrations. Data were expressed as mean ± SEM,^*^p< 0.05, ^**^p< 0.01 versus control. The control group was DMSO solution with 0 μM of **7j**.

Mitochondria dysfunction plays an important role in inducing apoptosis in cancer cells. The loss of MMP (Δ*Ψ*_m_) has been indicated as an early hallmark of mitochondrial dysfunction in apoptotic cells [38]. The cationic dye JC-1 is an ideal MMP-sensitive probe, which can be used to detect the changes of MMP by flow cytometry. As shown in Figure 6, **7j** induced a doses-dependent increase in depolarized cell population from 3.24% of control to 15.21%, 25.60% and 59.96% at the concentrations of 2, 4 and 8 μM, respectively. The results indicated that the apoptosis of HepG2 cells induced by **7j** was associated with the intrinsic mitochondrial-mediated pathways.

#### 2.2.4. Compound **7j** Induces Oxidative Stress in HepG2 Cells

**Figure 7:**
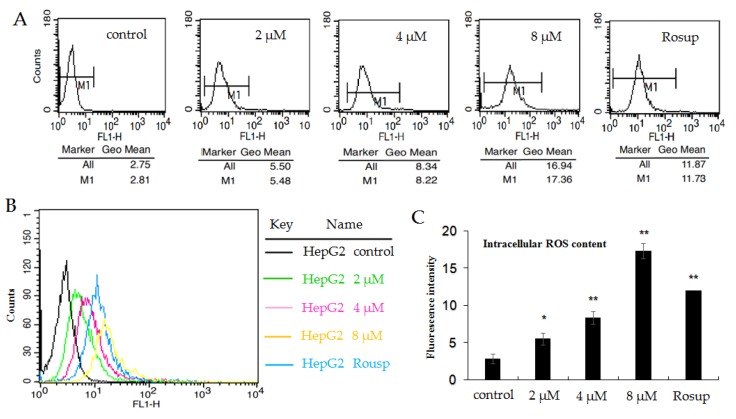
Effect of compound **7j** on ROS production in HepG2 cells. (**A**) HepG2 cells were treated with **7j** (2, 4 and 8 μM) for 24 h and stained with H2DCFDA, and then the cells were analyzed by flow cytometry. (**B**) Merge. (**C**) The bar graph represents the intracellular ROS content at various concentrations. Data were expressed as mean ± SEM, ^*^p< 0.05, ^**^p< 0.01 compared with the control.The control group was DMSO solution with 0 μM of **7j**.

Intracellular reactive oxygen species (ROS) plays a vital role in many kinds of biological processes. At the proper concentrations, ROS generation is considered as a major factor in mitochondrial-dependent apoptosis [39,40]. Hence, we explored the ROS inducing capability of **7j** at the cellular levels using 2’,7’-dichlorodihydrofluorescein diacetate (H2DCFDA) staining analysis by flow cytometry. As shown in Figure 7, we found a doses-dependent increase in the ROS generation was observed for HepG2 cells after treated with **7j** (2, 4 and 8 μM) for 24 h. The relative ROS level in HepG2 cells that were treated with **7j** (8 μM) was about six-fold higher than that of the control group, which was also apparently higher than the Rosup group. The results demonstrate that **7j** significantly increases ROS generation, which could be accountable for inducing apoptosis.

#### 2.2.5. Compound **7j** Induces Apoptosis Via the Activation of Caspases and Regulated Apoptosis Releated Protein Expression

**Figure 8:**
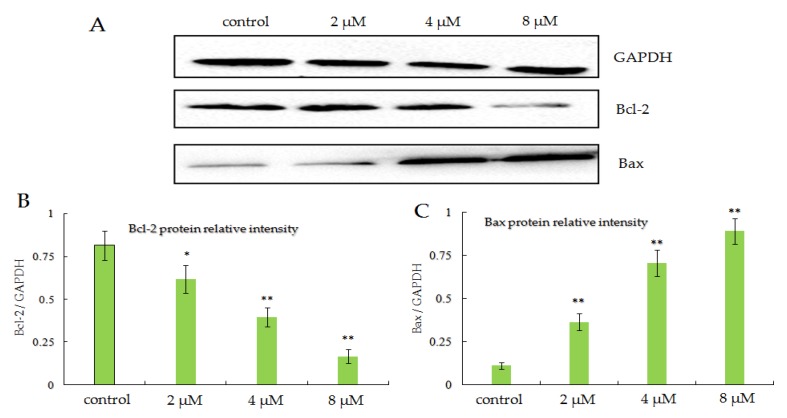
Effect of compound **7j** on the expression of apoptosis-related protein Bax and Bcl-2 in HepG2 cells was analyzed by Western blot. (**A**) Representative experiment. (**B**) The bar graph represents the Bcl-2 pretein relative intensity at various concentrations. (**C**) The bar graph represents the Bax pretein relative intensity at various concentrations. Data were expressed as mean ± SEM, ^*^p< 0.05, ^**^p< 0.01 versus control. The control group was DMSO solution with 0 μM of **7j**.

To further provide the molecular mechanistic insight into how compound **7j** induces apoptosis in HepG2 cells, we examined the expression of apoptotic proteins Bcl-2, Bax, caspase-3 and caspase-9 in response to compound **7j** treatment. First, HepG2 cells were treated with compound **7j** at the concentrations of 2, 4 and 8 µM for 24 h, and the expression levels of pro-apoptosis proteins Bax and pro-survival protein Bcl-2 were investigated by western blotting. The GAPDH expression was served as an internal control groups. As shown in Figure 8, the immunoblot analysis indicated that compound **7j** significantly suppressed the levels of Bcl-2 expression, but increased the expression levels of Bax in a dose-dependent manner.

**Figure 9:**
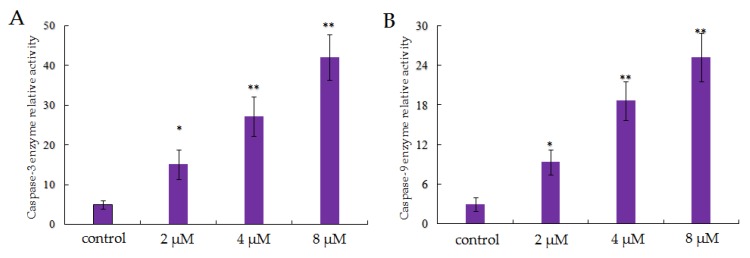
Effect of compound **7j** on the expression of casapse-3 and caspase-9 in HepG2 cells was analysed by spectrophotometry. (**A**) Relative activity of caspase-3; (**B**) Relative activity of caspase-9. Data were expressed as mean ± SEM, *p< 0.05, **p< 0.01 versus control. The control group was DMSO solution with 0 μM of **7j**.

Moreover, the expression levels of cleaved caspase-3 and caspase-9 were evaluated in HepG2 cells by spectrophotometry using caspase-3 and caspase-9 activity assay kits. As shown in Figure 9, the expression levels of cleaved caspase-3 and caspase-9 were significantly increased after being treated with **7j** (2, 4 and 8 µM), in a dose-dependent manner. The above data proved that compound **7j** induces HepG2 cells apoptosis via the intrinsic mitochondrial-mediated pathway.

## 3. Conclusions

In summary, we have successfully prepared a series of novel 5α,8α-endoperoxidesteroidal derivatives with semicarbazone/thiosemicarbazone side-chain on the C-17 position. The anti-proliferative activity of the compounds against HepG2, HCT-116, MCF-7 and A549cell lines were evaluated using the MTT assay *in vitro*. The results showed that compound **7j** exhibited significant cytotoxic activity against HepG2 cells (IC_50_ = 3.52 μM), and was more potent than ergosterol peroxide. Further cellular mechanism studies in HepG2 cells indicated that compound **7j** triggered the mitochondrial-mediated apoptosis by decreasing mitochondrial membrane potential (MMP), which was associated with up-regulation of Bax, down-regulation of Bcl-2, activation levels of the caspase cascade, and formation of reactive oxygen species (ROS). The above findings indicated that compound **7j** may be used as a promising skeleton for antitumor agents with improved efficacy.

## 4. Materials and Methods

### 4.1. Chemistry

Dehydroepiandrosterone, NBS, hydrazine hydrate, phloxine B, isocyanate, isothiocyanate substituents and other regents were purchased from Energy Chemical (Shanghai) Co. Ltd. China. All regents and solvent (analytical grade) used without further purification. ^1^H-NMR (600 MHz) and ^13^C-NMR (150 MHz) were recorded on a Bruker Avance DRX400 instrument (Bruker, Karlsruhe, Germany), using tetramethylsilane (TMS) as internal standards. Melting points (mp) were determined on an MP120 auto melting point apparatus (Haineng, Jinan, China). Mass spectra (ESI) of all compounds were recorded on Esquire 6000 mass spectrometer (Bruker, Karlsruhe, Germany). Flash chromatography was performed using 400 mesh silica gel.

#### 4.1.1. Synthesis of 3β-Acetoxyandrosta-5-en-17-one (**2**)

DHEA (**1**) (0.05 mol, 14.4 g) reacted with acetic anhydride (0.07 mol) in dichloromethane-pyridine (4:1, 100 mL). The reaction mixture was stirred at room temperature for 10 h. After completion of reaction, water (50 mL) was added into the mixture, and then extracted with dichloromethane (100 mL). The combined organic phase was washed with saturated NaHCO_3_ aqueous (2 × 60 mL), brine (2 × 60 mL) and dried over anhydrous Na_2_SO_4_. The solvent was evaporated and the crude product was purified by flash chromatography to obtain compound **2** as white solid (16.4 g, 97.7% yield). mp: 168.2–170 °C. ^1^H-NMR (600 MHz, CDCl_3_) *δ* 5.41 (d, *J* = 7.0 Hz, 1H, H-6), 4.60 (m, 1H), 2.45 (m, 1H), 2.33 (d, *J* = 7.6 Hz, 2H), 2.09 (m, 2H), 2.06 (s, 3H), 1.94 (d, *J* = 6.3 Hz, 1H), 1.88 (d, *J* = 4.6 Hz, 1H), 1.84 (m, 2H), 1.70–1.61 (m, 4H), 1.57 (m, 1H), 1.50 (m, 1H), 1.35–1.27 (m, 2H), 1.20–1.11 (m, 1H), 1.02 (s, 3H, 17-CH_3_), 1.01 (d, *J* = 3.8 Hz, 1H), 0.91 (s, 3H, 18-CH_3_). MS *m/z*: 353.8 [M+Na]^+^.

#### 4.1.2. Synthesis of 3β-Acetoxyandrosta-5,7-diene-17-one (**3**)

Intermediate **2** (0.05 mol, 16 g) reacted with NBS in cyclohexane (100 mL). The mixture was heated to 70 °C and refluxed for 1 h. The solids were removed by filtration, then the solvent was collected and evaporated to get a brown solid (17.0 g, 83%). Subsequently, the solid prepared above was dissolved in xylene (150 mL) and 2,4,6-collidine (25 mL). The mixture was heated to 135°C and refluxed for 3~4 h. After completion of reaction, the solids were removed by filtration. The solvent was collected and washed with water (2 ×80 mL), brine (2 ×80 mL) and dried over anhydrous Na_2_SO_4_. The solvent was evaporated and recrystallized in cooled methanol overnight, and then filtered to obtain pale yellow solid as compound **3** (5.6 g). Yield: 33%, mp: 111.8–114 °C; ^1^H-NMR (400 MHz, CDCl_3_) *δ* 5.60 (s, 1H), 5.57 (d, *J* = 4.8 Hz, 1H), 4.71 (m, 1H), 2.57–2.48 (m, 2H), 2.37 (d, *J* = 10.4 Hz, 1H), 2.26–2.16 (m, 2H), 2.09 (s, 3H), 1.99–1.93 (m, 2H), 1.91 (d, *J* = 4.6 Hz, 1H), 1.75 (d, *J* = 6.2 Hz, 2H), 1.68 (m, 1H), 1.60 (s, 2H), 1.39–1.32 (m, 2H), 1.28 (s, 1H), 1.00 (s, 3H), 0.85 (s, 3H). MS(ESI) *m*/*z*: 351.8 [M+Na]^+^.

#### 4.1.3. Synthesis of 3β-Hydroxyandrosta-5,7-diene-17-one (**4**)

Intermediate **3** (0.034 mol, 10.8 g) reacted with potassium hydroxide (4.5 g) in methanol (80 mL). The mixture was heated to 80 °C for 1 h. After completion of reaction, the mixture was cooled for crystallization overnight. The precipitate was washed with cooled methanol (20 mL) to get a brown solid crude product **4** (9.1 g). Yield: 93%, mp: 156.8–157.9 °C; ^1^H-NMR (400 MHz, CDCl_3_) *δ* 6.02 (d, *J* = 8.6 Hz, 1H), 5.70 (d, *J* = 9.4 Hz, 1H), 4.29 (m, 1H), 3.75–3.61 (m, 1H), 2.69–2.43 (m, 2H), 1.04 (s, 3H), 0.95 (s, 3H). MS (ESI) *m*/*z*: 310.0 [M+Na]^+^.

#### 4.1.4. Synthesis of 5α,8α-Cyclicobioxygen-6-vinyl-3β-ol-DHEA(**5**)

Intermediate **4** (3.24 mmol, 1.0 g) in methanol (150 mL) was added phloxine B (5 mg) in a 250 mL round-bottom flask. The mixture kept in a water-cooled bath and stirred by bubbling into high purity oxygen. While, the mixture was lighted with a 500 W iodine tungsten lamp (220 V) for 3 h. After completion of reaction, the solids were removed by filtration. The methanol was evaporated, and then the crude product was purified by chromatographic column (ethyl acetate/petroleum ether, 1:5) to give **5** as white needles (450.0 mg). Yield: 44%, m.p. 166.8–167.9 °C. ^1^H-NMR (600 MHz, CDCl_3_) *δ* 6.49 (d, *J* = 8.2 Hz, 1H), 6.35 (d, *J* = 8.0 Hz, 1H),3.97 (s, 1H), 2.60–2.49 (m, 1H), 2.25–2.11 (m,2H), 2.07–1.99 (m, 1H), 1.96 (m, 1H), 1.86 (m, 1H), 1.84–1.80 (m, 2H), 1.71 (m, 1H), 1.65–1.60 (m, 1H), 1.59–1.55 (m, 4H), 1.55–1.48 (m, 1H), 1.39–1.24 (m, 2H), 1.02 (s, 3H), 0.94 (s, 3H).^13^C-NMR (150 MHz, CDCl_3_) *δ* 217.9, 136.6, 130.0, 82.6, 78.8, 66.2, 52.0, 48.7, 47.6, 37.2, 36.7, 35.5, 34.8, 31.3, 29.8, 22.8, 19.0, 18.4, 15.1. MS (ESI) *m*/*z*: 319.2 [M+H]^+^.

#### 4.1.5. Synthesis of 5α,8α-Cyclicobioxygen-6-vinyl-3β-ol-DHEA(**6**)

Intermediate **5** (10.0 mmol, 3.0 g) reacted with 85% hydrazine hydrate (4 mL) in ethanol (100 mL). The mixture was heated to 40°C and stirred for 1~2 h. After completion of reaction, the solvent was evaporated, and then the crude product was purified by chromatographic column (ethyl acetate/petroleum ether, 1:1) to give **6** as white needles (2.7 g, 82%), m.p. 182.4–184.6 °C. ^1^H-NMR (600 MHz, DMSO-*d_6_*) *δ* 6.51 (d, *J* = 7.8 Hz, 1H, 6-H), 6.30 (d, *J* = 8.4 Hz, 1H, 7-H), 5.35 (d, *J* = 10.2 Hz, 2H, NH_2_), 4.66 (m, 1H, OH), 3.17 (m, 1H, 3-H), 1.00 (s, 3H, 18-CH_3_), 0.95 (s, 3H, 19-CH_3_). MS (ESI) *m*/*z*: 333.2 [M+H]^+^.

#### 4.1.6. General Procedure for Synthesis of Novel Derivatives **7a**–**k**

Intermediate **6** (1 mmol) reacted with phenyl isocyanate or isothiocyanate substituents (2 mmol) in the presence of ethanol (20 mL) and five drops of acetic acid for 5~8 h until no material. On completion of reaction, the solvent was evaporated by rotary evaporation. The residue was purified by flash chromatography (dichloromethane/methanol) to get pure target compounds **7a**–**k (**^1^H and ^13^C-NMR spectra of new compounds are in the Supplementary Materials).

*3β-Hydroxy-5α,8α-epidioxyandrost-17-N-(4-chlorophenyl)semicarbazide*(7**a**). Yellow powder, yield 88%, mp 138.3–140.0 °C. ^1^H-NMR (600 MHz, CDCl_3_) *δ* 8.07 (s, 1H, NH), 7.66 (s, 1H, NH), 7.44 (d, *J =* 8.5 Hz, 2H, Ar-H), 7.28 (d, *J =* 8.4 Hz, 2H, Ar-H), 6.49 (d, *J* = 8.5 Hz, 1H, 6-H), 6.32 (d, *J =* 8.4 Hz, 1H, 7-H), 3.99 (m, 1H, H-3), 2.49 (dd, *J =* 18.0, 8.8 Hz, 1H), 2.31 (dd, *J =* 17.7, 8.8 Hz, 1H), 2.14 (dd, *J =* 13.8, 3.7 Hz, 1H), 2.04–1.93 (m, 4H), 1.86–1.79 (m, 2H), 1.74–1.66 (m, 3H), 1.58 (dd, *J =* 13.3, 4.3 Hz, 2H), 1.51–1.45 (m, 1H), 1.32 (dd, *J =* 13.6, 3.2 Hz, 2H), 1.05 (s, 3H, 18-CH_3_), 0.93 (s, 3H, 19-CH_3_). ^13^C-NMR (150 MHz, CDCl_3_) δ161.3, 152.8, 136.6, 136.3, 129.8, 129.7, 128.9, 128.3, 120.6, 82.4, 78.7, 66.3, 51.5, 49.3, 46.0, 37.2, 36.8, 34.6, 34.1, 30.1, 29.7, 25.9, 22.9, 20.5, 18.4, 18.2.MS (ESI) *m*/*z*: [M+H]^+^ 486.2.

*3β-Hydroxy-5α,8α-epidioxyandrost-17-N-(3-(trifluoromethyl)phenyl)semicarbazide*(7b). Yellow powder, yield 90%, mp 145.4–146.8 °C. ^1^H-NMR (600 MHz, CDCl_3_) δ 8.53 (s, 1H, NH), 8.25 (s, 1H, NH), 7.88 (s, 1H, Ar-H), 7.61 (d, *J =* 7.4 Hz, 1H, Ar-H), 7.41 (t, *J =* 7.6 Hz, 1H, Ar-H), 7.30 (d, *J* = 7.5 Hz, 1H, Ar-H), 6.51 (d, *J =* 8.4 Hz, 1H, 6-H), 6.33 (d, *J =* 8.4 Hz, 1H, 7-H), 4.99 (m, 1H, H-3), 2.66–2.54 (m, 1H), 2.46–2.33 (m, 1H), 2.15 (dd, *J =* 13.6, 3.8 Hz, 1H), 1.99 (dd, *J =* 30.4, 12.7 Hz, 4H), 1.89–1.81 (m, 2H), 1.64–1.56 (m, 3H), 1.52 (d, *J =* 31.4 Hz, 2H), 1.32 (d, *J =* 13.5 Hz, 1H), 1.26 (m, 2H), 1.06 (s, 3H, 18-CH_3_), 0.93 (s, 3H, 19-CH_3_). ^13^C-NMR (150 MHz, CDCl_3_) δ 163.5, 138.7, 136.3, 129.8, 129.4, 122.2, 119.7, 115.9, 82.4, 78.8, 66.3, 51.5, 49.3, 46.0, 37.2, 36.8, 34.6, 34.2, 30.1, 29.7, 26.3, 22.9, 20.5, 18.4, 18.2.MS (ESI) *m*/*z*: [M+H]^+^ 520.2.

*3β-Hydroxy-5α,8α-epidioxyandrost-17-N-(4-methoxyphenyl)semicarbazide*(**7c**). Yellow powder, yield 88%, mp 130.3–132.0 °C. ^1^H-NMR (600 MHz, CDCl_3_) δ 7.96 (s, 1H, NH), 7.55 (s, 1H, NH), 7.39 (s, 2H, Ar-H), 6.87 (s, 2H, Ar-H), 6.50 (d, *J* = 8.1 Hz, 1H, 6-H), 6.32 (d, *J* = 8.1 Hz, 1H, 7-H), 3.98 (s, 1H, H-3), 3.79 (s, 3H, O-CH3), 2.48 (s, 1H), 2.31 (s, 1H), 2.14 (m, 1H), 2.05–1.92 (m, 4H), 1.85 (s, 2H), 1.74–1.66 (m, 3H), 1.57 (m, 2H), 1.47 (m, 1H), 1.26 (m, 2H), 1.05 (s, 3H, 18-CH_3_), 0.93 (s, 3H, 19-CH_3_). ^13^C-NMR (150 MHz, CDCl_3_) δ 156.0, 136.2, 129.8, 121.8, 114.2, 82.4, 78.8, 66.3, 55.5, 51.5, 49.3, 45.9, 37.1, 36.8, 34.6, 34.1, 30.1, 29.7, 22.9, 20.5, 18.5, 18.2. MS (ESI) *m*/*z*: [M+H]^+^ 482.2.

*3β-Hydroxy-5α,8α-epidioxyandrost-17-N-(m-tolyl)semicarbazide* (**7d**). Yellow powder, yield 86%, mp 155.3–156.8 °C.^1^H-NMR (600 MHz, DMSO-*d*_6_) δ 9.31 (s, 1H, NH), 8.42 (s, 1H, NH), 7.35 (d, *J* = 6.8 Hz, 2H, Ar-H), 7.15 (d, *J* = 8.5 Hz, 1H, Ar-H), 6.80 (d, *J* = 7.5 Hz, 1H, Ar-H), 6.49 (d, *J* = 8.5 Hz, 1H, 6-H), 6.28 (d, *J* = 8.4 Hz, 1H, 7-H), 4.62 (s, 1H), 3.59 (dd, *J* = 13.2, 7.9 Hz, 1H, H-3), 2.29 (m, 1H), 2.28 (s, 3H), 2.00 (m, 1H), 1.90–1.82 (m, 2H), 1.79–1.56 (m, 8H), 1.40 (m, 3H, Ar-CH3), 1.26–1.18 (m, 2H), 0.97 (s, 3H, 18-CH_3_), 0.83 (s, 3H, 19-CH_3_). ^13^C-NMR (150 MHz, DMSO-*d*_6_) δ 163.4, 153.8, 139.4, 138.3, 136.4, 130.3, 128.9, 123.4, 120.0, 116.7, 82.1, 78.7, 65.1, 51.7, 49.3, 46.0, 37.3, 37.1, 34.9, 34.1, 30.4, 26.9, 22.8, 21.6, 20.6, 18.6, 18.4.MS (ESI) *m*/*z*: [M+H]^+^ 466.2.

*3β-Hydroxy-5α,8α-epidioxyandrost-17-N-(4-(trifluoromethyl)phenyl)semicarbazide* (**7e**). Yellow powder, yield 89%, mp 138.4–140.1 °C. ^1^H-NMR (600 MHz, DMSO-*d*_6_) δ 9.50 (s, 1H, NH), 8.86 (s, 1H, NH), 7.80 (d, *J* = 8.5 Hz, 2H, Ar-H), 7.63 (d, *J* = 8.7 Hz, 2H, Ar-H), 6.49 (d, *J* = 8.5 Hz, 1H, 6-H), 6.28 (d, *J* = 8.5 Hz, 1H, 7-H), 4.62 (d, *J* = 5.0 Hz, 1H), 3.59 (dd, *J* = 10.2, 5.1 Hz, 1H, H-3), 2.3–2.24 (m, 1H), 2.03 (m, 1H), 1.87–1.82 (m, 2H), 1.81–1.54 (m, 8H), 1.42–1.34 (m, 3H), 1.25–1.17 (m, 2H), 0.98 (s, 3H, 18-CH3), 0.83 (s, 3H, 19-CH3). ^13^C-NMR (150 MHz, DMSO-*d*_6_) δ 164.3, 153.6, 143.3, 136.4, 130.3, 126.3, 124.1, 119.3, 82.1, 78.7, 65.1, 51.7, 49.3, 46.1, 37.3, 37.1, 34.9, 34.1, 30.4, 26.9, 22.8, 20.6, 18.5, 18.4.MS (ESI) *m*/*z*: [M+H]^+^ 520.2.

*3β-Hydroxy-5α,8α-epidioxyandrost-17-N-(4-chloro-3-(trifluoromethyl)phenyl)semicarbazide* (**7f**). Yellow powder, yield 85%, mp 127.5–129.0 °C. ^1^H-NMR (600 MHz, DMSO-*d*_6_) δ 9.52 (s, 1H, NH), 8.94 (s, 1H, NH), 8.18 (s, 1H, Ar-H), 7.92 (s, 1H, Ar-H), 7.61 (d, *J* = 7.1 Hz, 1H, Ar-H), 6.48 (d, *J* = 8.0 Hz, 1H, 6-H), 6.28 (d, *J* = 6.2 Hz, 1H, 7-H), 3.58 (m, 1H, H-3), 2.31 (m, 1H), 2.06 (m, 1H), 1.86 (m, 2H), 1.67 (m, 8H), 1.40 (m, 3H), 1.23 (m, 2H), 0.98 (s, 3H, 18-CH_3_), 0.83 (s, 3H, 19-CH_3_). ^13^C-NMR (150 MHz, DMSO-*d*_6_) δ 164.4, 154.2, 138.1, 136.4, 132.2, 130.3, 124.4, 123.6, 119.1, 82.1, 78.7, 65.1, 51.7, 49.3, 46.1, 37.1, 37.1, 34.9, 34.1, 30.4, 27.0, 22.8, 20.6, 18.5, 18.4.MS (ESI) *m*/*z*: [M+H]^+^ 554.2.

*3β-Hydroxy-5α,8α-epidioxyandrost-17-N-(4-cyanophenyl)semicarbazide* (**7g**). Yellow powder, yield 91%, mp 161.4–162.8 °C. ^1^H-NMR (600 MHz, DMSO-*d*_6_) δ 9.55 (s, 1H, NH), 8.94 (s, 1H, NH), 7.78 (d, *J* = 8.6 Hz, 2H, Ar-H), 7.72 (d, *J* = 8.7 Hz, 2H, Ar-H), 6.49 (d, *J* = 8.5 Hz, 1H, 6-H), 6.28 (d, *J* = 8.5 Hz, 1H, 7-H), 3.58 (dd, *J* = 10.0, 4.9 Hz, 1H, H-3), 2.30 (dd, *J* = 18.5, 9.1 Hz, 1H), 2.02 (d, *J* = 13.0 Hz, 1H), 1.89–1.83 (m, 2H), 1.80–1.54 (m, 8H), 1.47–1.34 (m, 3H), 1.30–1.11 (m, 2H), 1.06 (m, 1H), 0.97 (s, 3H, 18-CH_3_), 0.83 (s, 3H, 19-CH_3_). ^13^C-NMR (150 MHz, DMSO-*d*_6_) δ 164.6, 144.1, 136.4, 133.5, 130.3, 119.4, 104.1, 82.1, 78.7, 65.1, 51.7, 49.2, 46.1, 37.3, 37.1, 34.9, 34.1, 30.4, 27.0, 22.8, 20.5, 18.5, 18.4.MS (ESI) *m*/*z*: [M+H]^+^ 477.2.

*3β-Hydroxy-5α,8α-epidioxyandrost-17-N-(4-chlorophenyl)thiosemicarbazide* (**7h**). Yellow powder, yield 93%,mp 166.2–168.0 °C. ^1^H-NMR (600 MHz, CDCl_3_) δ 9.11 (s, 1H, NH), 8.39 (s, 1H, NH), 7.59 (m, 2H, Ar-H), 7.35 (m, 2H, Ar-H), 6.49 (d, *J* = 8.5 Hz, 1H, 6-H), 6.33 (d, *J* = 8.5 Hz, 1H, 7-H), 4.00–3.92 (m, 1H, H-3), 2.55 (m, 1H), 2.45–2.34 (m, 1H), 2.14 (m, 1H), 2.05–1.94 (m, 4H), 1.83 (m, 2H), 1.76–1.64 (m, 4H), 1.59–1.53 (m, 2H), 1.48 (m, 1H), 1.35–1.24 (m, 2H), 1.06 (s, 3H, 18-CH3), 0.92 (s, 3H, 19-CH3). ^13^C-NMR (150 MHz, CDCl^3^) δ 176.3, 164.9, 136.4, 131.3, 129.6, 128.8, 125.5, 82.4, 78.7, 66.2, 51.5, 49.2, 46.3, 37.1, 36.7, 34.7, 34.1, 30.1, 26.2, 22.8, 20.5, 18.4, 18.2.MS (ESI) *m*/*z*: [M+H]^+^ 502.2.

*3β-Hydroxy-5α,8α-epidioxyandrost-17-N-(4-methoxyphenyl)thiosemicarbazide* (**7i**). Yellow powder, yield 90%, mp 157.4–159.1 °C. ^1^H-NMR (600 MHz, CDCl_3_) δ 8.98 (s, 1H, NH), 8.32 (s, 1H, NH), 7.45 (d, *J* = 8.9 Hz, 2H, Ar-H), 6.93 (d, *J* = 3.1 Hz, 2H, Ar-H), 6.49 (d, *J* = 8.5 Hz, 1H, 6-H), 6.33 (d, *J* = 8.5 Hz, 1H, 7-H), 4.01–3.93 (m, 1H, H-3), 3.82 (s, 3H, O-CH3), 2.54 (m, 1H), 2.45–2.34 (m, 1H), 2.17–2.10 (m, 1H), 2.08–1.92 (m, 4H), 1.84 (m, 2H), 1.77–1.70 (m, 2H), 1.69–1.63 (m, 2H), 1.61–1.52 (m, 2H), 1.51–1.38 (m, 1H), 1.29 (m, 2H), 1.06 (s, 3H, 18-CH_3_), 0.92 (s, 3H, 19-CH_3_). ^13^C-NMR (150 MHz, CDCl_3_) δ 177.1, 164.3, 157.9, 136.4, 130.8, 129.6, 126.6, 114.0, 82.4, 78.7, 66.2, 55.5, 51.6, 49.2, 46.2, 37.1, 36.7, 34.7, 34.1, 30.1, 26.1, 22.9, 20.6, 18.4, 18.2.MS (ESI) *m*/*z*: [M+H]^+^ 497.2.

*3β-Hydroxy-5α,8α-epidioxyandrost-17-N-(3-chlorophenyl)thiosemicarbazide* (**7j**). Yellow powder, yield 86%, mp 166.5–168.1 °C. ^1^H-NMR (600 MHz, CDCl_3_) δ 9.15 (s, 1H, NH), 8.35 (s, 1H, NH), 7.71 (s, 1H, Ar-H), 7.59 (d, *J* = 7.9 Hz, 1H, Ar-H), 7.34 (d, *J* = 3.2 Hz, 1H, Ar-H), 7.20 (d, *J* = 7.9 Hz, 1H, Ar-H), 6.49 (d, *J* = 8.4 Hz, 1H, 6-H), 6.33 (d, *J* = 8.4 Hz, 1H, 7-H), 4.08–3.90 (m, 1H, H-3), 2.56 (m, 1H), 2.42–2.29 (m, 1H), 2.14 (dd, *J* = 13.7, 4.4 Hz, 1H), 2.07–1.92 (m, 4H), 1.90–1.81 (m, 2H), 1.63 (m, 6H), 1.36–1.23 (m, 2H), 1.07 (s, 3H, 18-CH_3_), 0.93 (s, 3H, 19-CH_3_). ^13^C-NMR (150 MHz, CDCl_3_) δ 176.1, 164.8, 139.1, 136.4, 134.2, 129.7, 129.6, 126.0, 123.9, 122.1, 82.4, 78.7, 66.3, 51.5, 49.2, 46.3, 37.1, 36.8, 34.7, 34.1, 30.1, 26.2, 22.8, 20.6, 18.4, 18.2.MS (ESI) *m*/*z*: [M+H]^+^ 502.2.

*3β-Hydroxy-5α,8α-epidioxyandrost-17-N-(p-tolyl)thiosemicarbazide* (**7k**). Yellow powder, yield 90%, mp 151.2–153.0 °C. ^1^H-NMR (600 MHz, DMSO-*d*_6_) δ 10.27 (s, 1H, NH), 9.57 (s, 1H, NH), 7.41 (d, *J* = 8.1 Hz, 2H, Ar-H), 7.14 (d, *J* = 8.1 Hz, 2H, Ar-H), 6.50 (d, *J* = 8.5 Hz, 1H, 6-H), 6.28 (d, *J* = 8.4 Hz, 1H, 7-H), 3.59 (dd, *J* = 9.8, 4.6 Hz, 1H, H-3), 2.75–2.64 (m, 1H), 2.43 (m, 1H), 2.29 (s, 3H), 2.01 (m, 1H), 1.84–1.56 (m, 8H), 1.47–1.30 (m, 3H, Ar-CH_3_), 1.23 (s, 2H), 0.98 (s, 3H, 18-CH_3_), 0.82 (s, 3H, 19-CH_3_). ^13^C-NMR (150 MHz, DMSO-*d*_6_) δ 176.9, 172.5, 166.8, 136.8, 136.4, 134.7, 130.3, 129.0, 125.5, 82.1, 78.7, 65.1, 51.7, 49.1, 46.5, 37.3, 37.1, 34.9, 33.9, 30.4, 27.4, 22.8, 21.5, 21.0, 20.6, 18.4, 18.3. MS (ESI) *m*/*z*: [M+H]^+^ 482.2.

### 4.2. Biological Evaluation

#### 4.2.1. Cell Culture

HepG2, MCF-7, A549 and HCT-116 cancer cell lines were obtained from Qiqihar Medical University. Cells were cultured in DMEM medium supplemented with 10% FBS, 100 units/mL penicillin and 100 μg/mL streptomycin at 37 °C in a humidified atmosphere of 5% CO_2_. All reagents were purchased from HyClone (Logan, UT, USA).

#### 4.2.2. MTT Assay

Cytotoxic activities of all synthesized compounds against HepG2, MCF-7, A549 and HCT-116 cancer cell lines were tested by the MTT assay. Cells were seeded into 96-well plates (1×10^4^ cells/well) for 24 h. Then the cells were treated with compounds at gradient concentrations from 2μM to 60 μM for 48 h and then 10 μL MTT (Sigma Chemical Co., Ltd., Milwaukee, WI, USA) solution (5 mg/mL in PBS) were added for 2 h. The solution was replaced by 100 μL DMSO, and the absorbance was measured at 490 nm on a Spectra Max 340 microplate reader. The IC_50_values were derived by SPSS nonlinear regression analysis.

#### 4.2.3. Determination of Morphological Changes of Cells

The morphological changes of cells were tested with Hoechst 33342/PI double stain kit (Solarbio, Basingstoke, England). HepG2 cells were seeded in 6-well plates (2 ×10^5^ cells/well). After stabilization for 24 h, the cells were treated with compound **7j** (0, 2, 4 and 8 μM) for 48 h. Then the cells were fixed with 4% formaldehyde for 1 h at 4°C, and the cells were treated with 5 μL Hoechst33342 and 5 μL PI at 37°C in the dark for 20 min. Then the morphological changes of HepG2 cells were observed using a fluorescence microscope [40].

#### 4.2.4. Apoptosis Analysis by Flow Cytometry

Cell apoptosis analysis was detected by an Annexin V-FITC/PI apoptosis detection kit (Beyotime, Shanghai, China). HepG2 cells were seeded into 6-well plates at a concentration of 7 × 10^4^ cells/mL for overnight. The cells were treated with compound **7j** (0, 2, 4 and 8 μM) for 24 h. The cells were collected, washed twice with PBS. The cells were resuspended in the Annexin V-FITC/PI staining solution for 15 min. The cells apoptosis rates were analyzed by flow cytometry (BD Biosciences, San Jose, CA, USA) [41].

#### 4.2.5. Analysis of Mitochondrial Membrane Potential

HepG2 cells were seeded in a 12-well plate (1 × 10^6^ cells/well). Then cells were treated with different doses of **7j** (0, 2, 4 and 8 µM) for 24 h. After treatment, cells were treated with 5 µM cationic dye JC-1 (KGA601, Nanjing KeyGEN Biotech, Nanjing, China) for 30 min. Finally, cells were harvested and washed with PBS, and then analyzed by flow cytometer (BD Biosciences, San Jose, CA, USA) [42].

#### 4.2.6. Measurement of ROS by Flow Cytometry

HepG2 cells were seeded in 12-well plate (1 × 10^6^ cells/well) for 24 h. Then cells were treated with different doses of **7j** (0, 2, 4 and 8 µM) for 24 h. After treatment, 400 µL of H2DCFDA solution (5 µM) was added to per well and incubated for 30 min. Then, removed the H2DCFDA solution and trypsinised the cells, and centrifuged. Washed the obtained pellet with PBS and centrifuged for 3 min. A last, of PBS (400 µL) was added and analyzed by flow cytometer (BD Biosciences, San Jose, CA, USA) [43].

#### 4.2.7. Western Blot Analysis

After treatment with compound **7j** (0, 2, 4 and 8 µM), HepG2 cells were harvested with trypsin and lysed in RIPA buffer and boiled at 100 °C for 10 min. Protein was separated on a 15% SDS-PAGE gel, and transferred to nitrocellulose membranes. The membranes were blocked with 5% BSA for 1~2 h, and incubated with a 1:1000 dilution of primary antibody overnight. Then the membranes were incubated with a 1:5000 dilution of secondary antibody for 2 h. Positive bands were visualized by using an enhanced chemiluminescence system Kit [44].

## Figures and Tables

**Table 1:** *In vitro* anti-proliferativeactivities of compounds**7a**–**k** in human cancer cells.

Compound	X	R	IC_50_ (μM) ^a^			
			HepG2	HCT-116	MCF-7	A549
**7a**	O	4-Cl	8.65 ±0.72	32.59 ± 2.18	19.31 ± 1.08	21.43 ± 1.12
**7b**	O	3-CF_3_	5.73 ± 0.78	10.48 ± 0.59	8.62 ± 0.71	18.04 ± 0.93
**7c**	O	4-OCH_3_	>60	>60	52.32 ± 2.14	>60
**7d**	O	4-CH_3_	37.50 ± 2.26	>60	>60	47.33 ± 2.17
**7e**	O	4-CF_3_	7.51 ± 0.77	26.44 ± 1.43	14.90 ± 0.90	21.88 ± 1.21
**7f**	O	3-CF_3_-4-Cl	4.34 ± 0.41	12.78 ± 1.42	7.37 ± 0.42	6.03 ± 0.81
**7g**	O	4-CN	6.06 ± 0.69	14.07 ± 1.58	8.98 ± 0.70	16.93 ± 1.77
**7h**	S	4-Cl	5.44 ± 0.42	15.41 ± 1.63	7.66 ± 0.49	11.33 ± 0.69
**7i**	S	4-OCH_3_	14.35± 0.82	>60	26.73 ± 1.75	>60
**7j**	S	3-Cl	3.52± 0.35	9.79 ± 0.55	4.88 ± 0.42	7.70 ± 0.62
**7k**	S	4-CH_3_	19.07 ± 1.09	48.36 ± 4.43	25.73 ± 1.52	>60
EP	-	-	21.35± 1.13	26.42± 1.32	16.04 ± 0.81	19.48 ± 1.16
Mitomycin	-	-	29.36 ± 1.14	10.52± 0.87	16.56± 1.09	12.47 ± 1.04

^a^ IC_50_: Concentration of the tested compound that inhibits 50% of cell growth. All data are presented as means ± SD of three independent experiments.

## Supplementary Materials

The following are available online at https://www.mdpi.com/1420-3049/25/5/1209/s1, Supplementary Materials for ^1^H and ^13^C-NMR spectra of new compounds are available online.

## References

[1] Zhang M.M., Qiao Y., Ang E.L., Zhao. H. (2017). Using natural products for drug discovery: The impact of the genomics era. Expert. Opin. Drug. Discov..

[2] Rodrigues T., Reker D., Schneider P., Schneider G. (2016). Counting on natural products for drug design. Nat. Chem..

[3] Bu M., Yang B.B., Hu L.M. (2016). Natural endoperoxides as drug lead compounds. Curr. Med. Chem..

[4] Jung M., Kim H., Lee K., Park M. (2003). Naturally occurring peroxides with biological activities. Mini-Rev. Med. Chem..

[5] Li H., Huang H., Shao C., Huang H., Jiang J., Zhu X., Liu Y., Liu L., Lu Y., Li M. (2011). Cytotoxic norsesquiterpene peroxides from the endophytic fungus *Talaromyces flavus* isolated from the mangrove plant *Sonneratia apetala*. J. Nat. Prod..

[6] Imamura Y., Yukawa M., Ueno M., Kimura K.I., Tsuchiya E. (2014). 3,6-Epidioxy-1,10-bisaboladiene inhibits G1 -specific transcription through Swi4/Swi6 and Mbp1/Swi6 via the Hog1 stress pathway in yeast. FEBS J..

[7] Dembitsky V.M., Gloriozova T.A., Poroikov V.V. (2007). Natural peroxy anticancer agents. Mini-Rev. Med. Chem..

[8] Zhang C.C., Yin X., Cao C.Y., Wei J., Zhang Q., Gao J.M. (2015). Chemical constituents from Hericium erinaceus and their ability to stimulate NGF-mediated neurite outgrowth on PC12 cells. Bioorg. Med. Chem. Lett..

[9] Callaway E., Cyranoski D. (2015). Anti-parasite drugs sweep Nobel prize in medicine 2015. Nature.

[10] Lam N.S., Long X., Su X.Z., Lu F. (2018). Artemisinin and its derivatives in treating helminthic infections beyond schistosomiasis. Pharmacol. Res..

[11] Slezakova S., Ruda-Kucerova J. (2017). Anticancer Activity of Artemisinin and its derivatives. Anticancer Res..

[12] Cui J.G., Liu L., Zhao D.D., Gan C.F., Huang X., Xiao Q., Qi B.B., Yang L., Huang Y.M. (2015). Synthesis, characterization and antitumor activities of some steroidal derivatives with side chain of 17-hydrazone aromatic heterocycle. Steroids.

[13] Ke S.Y., Wei Y.H., Shi L.Q., Yang Q.Y., Yang Z.W. (2013). Synthesis of novel steroid derivatives derived from dehydroepiandrosterone as potential anticancer agents. Anticancer Agents Med. Chem..

[14] Yu B., Zhang E., Sun X.N., Ren J.L., Fang Y., Zhang B.L., Yu D.Q., Liu H.M. (2013). Facile synthesis of novel D-ring modified steroidal dienamides via rearrangement of 2H-pyrans. Steroids.

[15] Zhang J., Wang X., Yang J., Guo L., Wang X., Song B., Dong W., Wang W. (2020). Novel diosgenin derivatives containing 1,3,4-oxadiazole/thiadiazole moieties as potential antitumor agents: Design, synthesis and cytotoxic evaluation. Eur. J. Med. Chem..

[16] Hanson J.R. (2010). Steroids: Partial synthesis in medicinal chemistry. Nat. Prod. Rep..

[17] Jeong Y.U., Park Y.J. (2020). Ergosterol peroxide from the medicinal mushroom *Ganoderma lucidum* inhibits differentiation and lipid accumulation of 3T3-L1 adipocytes. Int. J. Mol. Sci..

[18] He L., Shi W., Liu X., Zhao X., Zhang Z. (2018). Anticancer action and mechanism of ergosterol peroxide from *paecilomyces cicadae* fermentation broth. Int. J. Mol. Sci..

[19] Nowak R., Drozd M., Mendyk E., Lemieszek M., Krakowiak O., Kisiel W., Rzeski W., Szewczyk K. (2016). A new method for the isolation of ergosterol and peroxy ergosterol as active compounds of hygrophoropsis aurantiaca and *in*
*vitro* antiproliferative activity of isolated ergosterol peroxide. Molecules.

[20] Wu Q.P., Xie Y.Z., Deng Z., Li X.M., Yang W., Jiao C.W., Fang L., Li S.Z., Pan H.H., Yee A.J. (2012). Ergosterol peroxide isolated from *Ganoderma lucidum* abolishes microRNA miR-378-mediated tumor cells on chemoresistance. PLoS ONE.

[21] Li X.M., Wu Q.P., Bu M., Hu L.M., Du W.W., Jiao C.W., Pan H.H., Sdiri M., Wu N., Xie Y.Z. (2016). Ergosterol peroxide activates Foxo3-mediated cell death signaling by inhibiting AKT and c-Myc in human hepatocellular carcinoma cells. Oncotarget.

[22] Bu M., Cao T.T., Li H.X., Guo M.Z., Yang B.B., Zhou Y., Zhang N., Zeng C.C., Hu L.M. (2017). Synthesis and biological evaluation of novel steroidal 5α,8α-endoperoxide derivatives with aliphatic side-chain as potential anticancer agents. Steroids.

[23] Bu M., Cao T.T., Li H.X., Guo M.Z., Yang B.B., Zeng C.C., Hu L.M. (2017). Synthesis of 5α,8α-ergosterol peroxide 3-carbamate derivatives and a fluorescent mitochondria-targeting conjugate for enhanced anticancer activities. ChemMedChem.

[24] Bu M., Li H., Wang H., Wang J., Lin Y., Ma Y. (2019). Synthesis of ergosterol peroxide conjugates as mitochondria targeting probes for enhanced anticancer activity. Molecules.

[25] Bu M., Cao T., Li H., Guo M., Yang B.B., Zeng C., Zhou Y., Zhang N., Hu L. (2017). Synthesis and biological evaluation of novel steroidal 5α,8α-epidioxyandrost-6-ene-3β-ol-17-(*O*-phenylacetamide)oxime derivatives as potential anticancer agents. Bioorg. Med. Chem. Lett..

[26] Wang H.J., Bu M., Wang J., Liu L., Zhang S. (2019). Synthesis and biological evaluation of novel steroidal 5α, 8α-endoperoxide steroidal derivatives with aromatic hydrazone side chain as potential anticancer agents. Russ. J. Bioorg. Chem..

[27] Li H.L., Wang H.J., Wang J., Lin Y., Ma Y., Bu M. (2020). Design, synthesis and biological evaluation of novel 5α, 8α-endoperoxide steroidal derivatives with hybrid side chain as anticancer agents. Steroids.

[28] Qazi S.U., Rahman S.U., Awan A.N., Al-Rashida M., Alharthy R.D., Asari A., Hameed A., Iqbal J. (2018). Semicarbazone derivatives as urease inhibitors: Synthesis, biological evaluation, molecular docking studies and in-silico ADME evaluation. Bioorg. Chem..

[29] Sinniah S.K., Tan K.W., Ng S.W., Sim K.S. (2017). Thiosemicarbazone derivative induces in vitro apoptosis in metastatic PC-3 cells via activation of mitochondrial pathway. Anticancer Agents Med. Chem..

[30] Xu H., Su X., Liu X.Q., Zhang K.P., Hou Z., Guo C. (2019). Design, synthesis and biological evaluation of novel semicarbazone-selenochroman-4-ones hybrids as potent antifungal agents. Bioorg. Med. Chem. Lett..

[31] Cavalcanti de Queiroz A., Alves M.A., Barreiro E.J., Lima L.M., Alexandre-Moreira M.S. (2019). Semicarbazone derivatives as promising therapeutic alternatives in leishmaniasis. Exp. Parasitol..

[32] Demoro B., Bento-Oliveira A., Marques F., Costa Pessoa J., Otero L., Gambino D., F M de Almeida R., Tomaz A.I. (2019). Interaction with blood proteins of a ruthenium (II) nitrofuryl semicarbazone complex: Effect on the antitumoral activity. Molecules.

[33] Ma J., Ni X., Gao Y., Huang K., Wang Y., Liu J., Gong G. (2019). Semicarbazone derivatives bearing phenyl moiety: Synthesis, anticancer activity, cell cycle, apoptosis-inducing and metabolic stability study. Chem. Pharm. Bull..

[34] Palanimuthu D., Poon R., Sahni S., Anjum R., Hibbs D., Lin H.Y., Bernhardt P.V., Kalinowski D.S., Richardson D.R. (2017). A novel class of thiosemicarbazones show multi-functional activity for the treatment of Alzheimer’s disease. Eur. J. Med. Chem..

[35] Pham V.H., Phan T.P.D., Phan D.C., Vu B.D. (2020). Synthesis and bioactivity of thiosemicarbazones containing adamantane skeletons. Molecules.

[36] Hałdys K., Goldeman W., Jewgiński M., Wolińska E., Anger-Góra N., Rossowska J., Latajka R. (2020). Halogenated aromatic thiosemicarbazones as potent inhibitors of tyrosinase and melanogenesis. Bioorg. Chem..

[37] Khan S.A., Asiri A.M. (2018). Multi-step synthesis, spectroscopic studies of biological active steroidal thiosemicarbazones and their palladium (II) complex as macromolecules. Int. J. Biol.Macromol..

[38] Jabeen M., Choudhry M.I., Miana G.A., Rahman K.M., Rashid U., Khan H.U., Sadiq A. (2018). Synthesis, pharmacological evaluation and docking studies of progesterone and testosterone derivatives as anticancer agents [J]. Steroids.

[39] Yadav P., Lal K., Kumar A., Guru S.K., Jaglan S., Bhushan S. (2017). Green synthesis and anticancer potential of chalcone linked-1,2,3-triazoles. Eur. J. Med. Chem..

[40] Weidner C., Rousseau M., Plauth A., Wowro S.J., Fischer C., Abdel-Aziz H., Sauer S. (2016). Iberisamara extract induces intracellular formation of reactive oxygen species and inhibits colon cancer. PLoS ONE.

[41] Swanepoel B., Nitulescu G.M., Olaru O.T., Venables L., van de Venter M. (2019). Anti-cancer activity of a 5-aminopyrazole derivative lead compound (BC-7) and potential synergistic cytotoxicity with cisplatin against human cervical cancer cells. Int. J. Mol. Sci..

[42] Huang X., Wang M., Wang C., Hu W., You Q., Yang Y., Yu C., Liao Z., Gou S., Wang H. (2019). Dual-targeting antitumor conjugates derived from platinum (IV) prodrugs and microtubule inhibitor CA-4 significantly exhibited potent ability to overcome cisplatin resistance. Bioorg. Chem..

[43] Prabhu K.S., Siveen K.S., Kuttikrishnan S., Jochebeth A., Ali T.A., Elareer N.R., Iskandarani A., Quaiyoom Khan A., Merhi M., Dermime S. (2019). Greensporone A, a fungal secondary metabolite suppressed constitutively activated AKT via ROS generation and induced apoptosis in leukemic cell lines. Biomolecules.

[44] Cui D., Zhang C., Liu B., Shu Y., Du T., Shu D., Wang K., Dai F., Liu Y., Li C. (2015). Regression of gastric cancer by systemic injection of RNA nanoparticles carrying both ligand and siRNA. Sci. Rep..

