# Supplementary material for: Novel Steroidal 5α,8α-Endoperoxide Derivatives with Semicarbazone/Thiosemicarbazone Side-chain as Apoptotic Inducers through an Intrinsic Apoptosis Pathway: Design, Synthesis and Biological Studies
Source: Molecules. 2020 Mar 7;25(5):1209. doi: 10.3390/molecules25051209 (PMC7179397; doi:10.3390/molecules25051209)
Supplement: Supplementary file 1 [file molecules-25-01209-s001.pdf]

Supplementary Data

Novel Steroidal 5α,8α-Endoperoxide Derivatives with Semicarbazone/Thiosemicarbazone Side-chain as Apoptotic Inducers through an Intrinsic Apoptosis Pathway: Design, Synthesis and Biological Studies

Liwei Ma[1], Haijun Wang[2], Jing Wang[2], Lei Liu[2], Song Zhang[3], and Ming Bu[2,*]

[1] Research Institute of Medicine & Pharmacy, Qiqihar Medical University, Qiqihar 161006, China;

[2] College of Pharmacy, Qiqihar Medical University, Qiqihar 161006, China;

[3] Basic Medical Science College, Qiqihar Medical University, Qiqihar 161006, China.

List of contents:

^{1}H NMR and ^{13}C NMR spectrum of **7a**……………………………………………………..…S2
^{1}H NMR and ^{13}C NMR spectrum of **7b**……………………………………………………..…S3
^{1}H NMR and ^{13}C NMR spectrum of **7c**……………………………………………………..…S4
^{1}H NMR and ^{13}C NMR spectrum of **7d**……………………………………………………..…S5
^{1}H NMR and ^{13}C NMR spectrum of **7e**……………………………………………………..…S6
^{1}H NMR and ^{13}C NMR spectrum of **7f**……………………………………………………..…S7
^{1}H NMR and ^{13}C NMR spectrum of **7g**……………………………………………………..…S8
^{1}H NMR and ^{13}C NMR spectrum of **7h**……………………………………………………..…S9
^{1}H NMR and ^{13}C NMR spectrum of **7i**……………………………………………………..…S10
^{1}H NMR and ^{13}C NMR spectrum of **7j**……………………………………………………..…S11
^{1}H NMR and ^{13}C NMR spectrum of **7k** ……………………………………………………..…S12

* Correspondence: buming@qmu.edu.cn (M.B.); Tel.: +86-0452-2663-881

Compound 7a

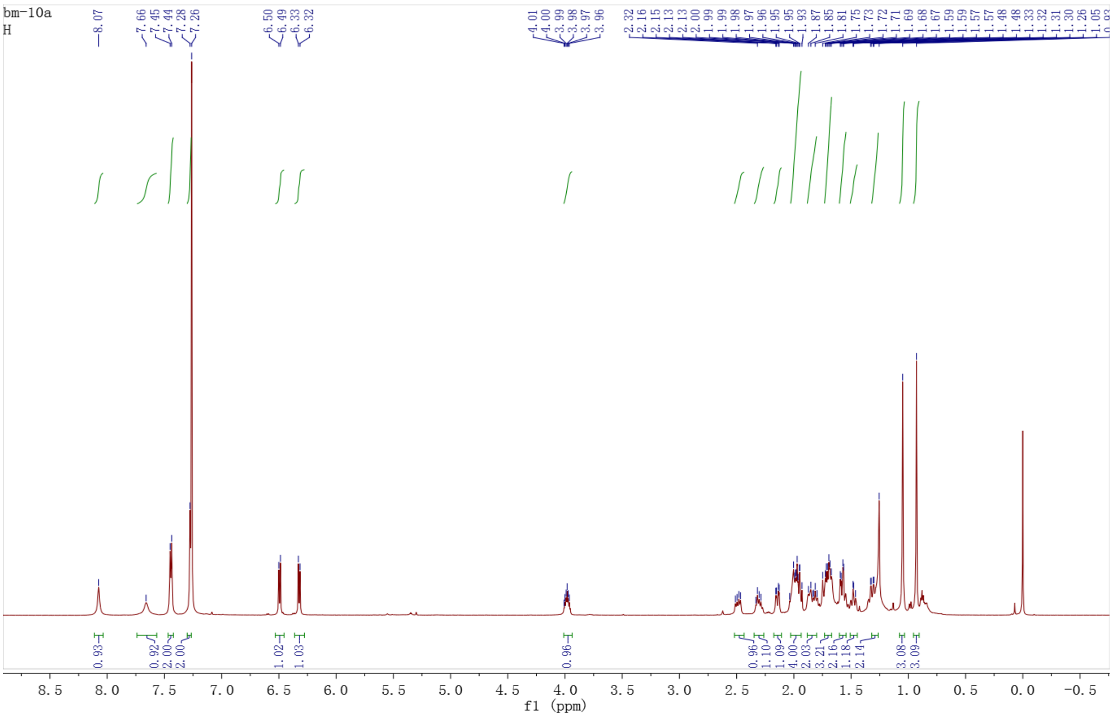
bm-10a
H
8.07
7.66
7.45
7.44
7.28
7.26
6.50
6.49
6.33
6.32
4.01
4.00
3.99
3.98
3.97
3.96
0.93
0.92
2.00
2.00
1.02
1.03
0.96
0.96
1.10
1.09
4.00
2.03
3.21
2.16
1.18
2.14
3.08
3.09
f1 (ppm)

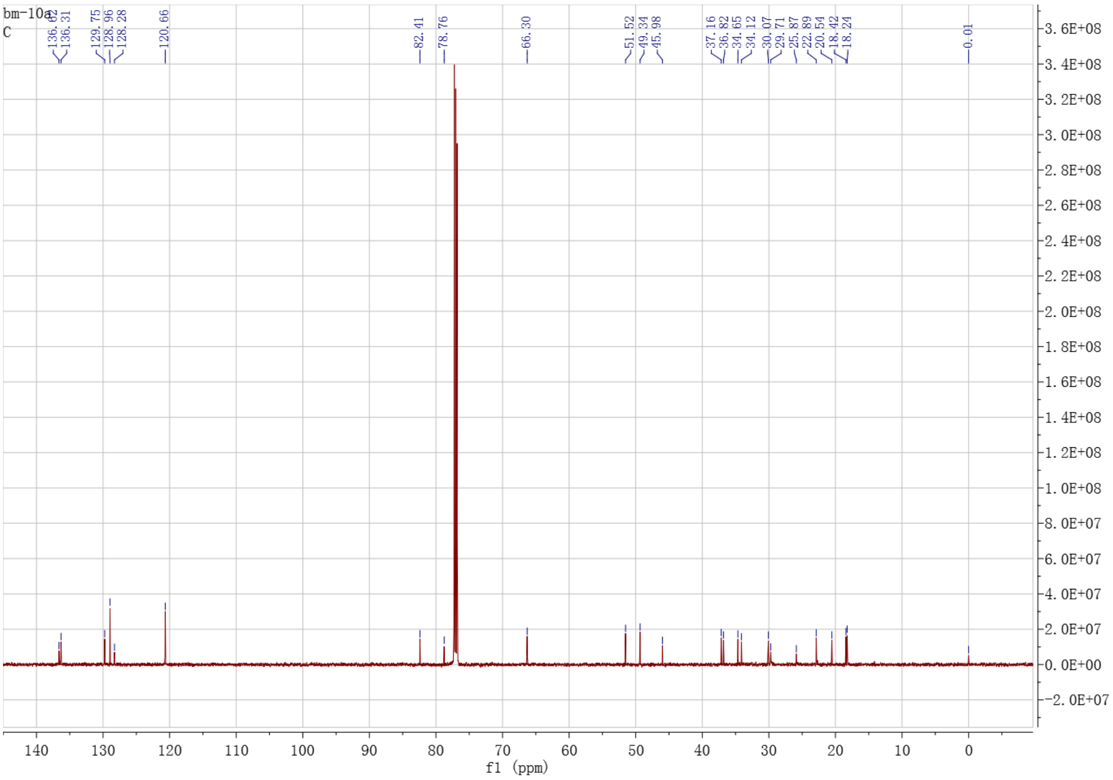
bm-10a
C
136.31
129.75
128.96
128.28
120.66
82.41
78.76
66.30
51.52
49.34
45.98
37.16
36.82
34.65
34.12
30.07
29.71
25.87
22.89
20.54
18.42
18.24
0.01
f1 (ppm)

Compound 7b

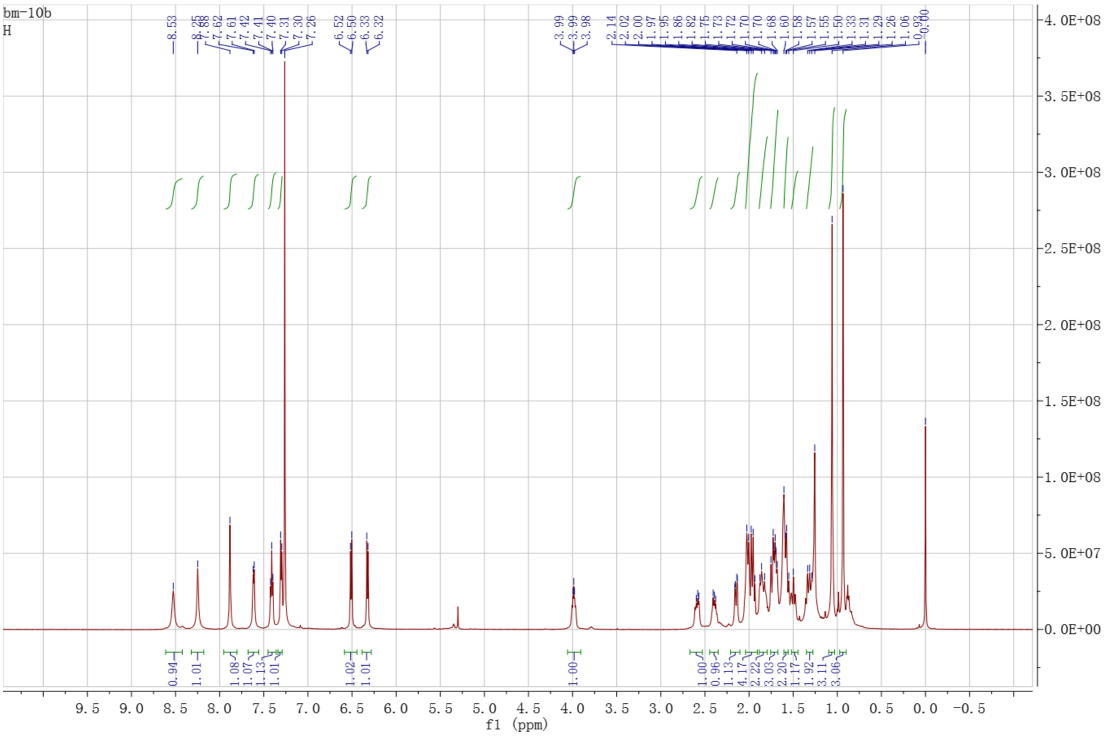

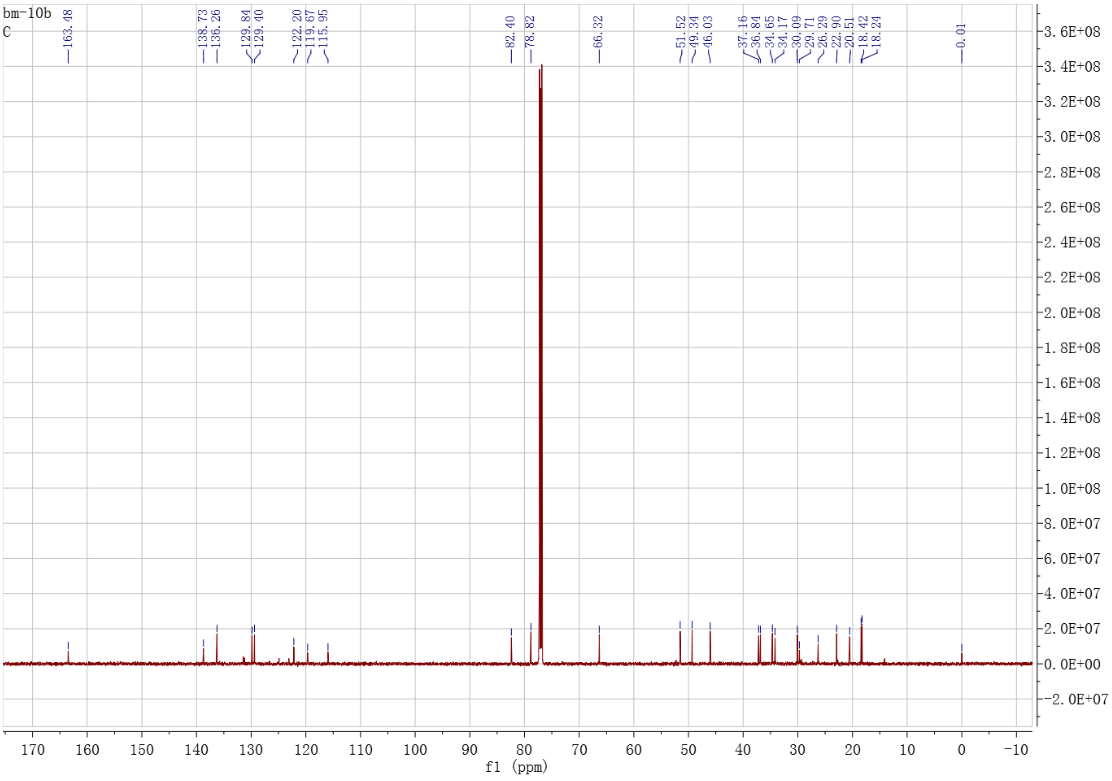

Compound 7c

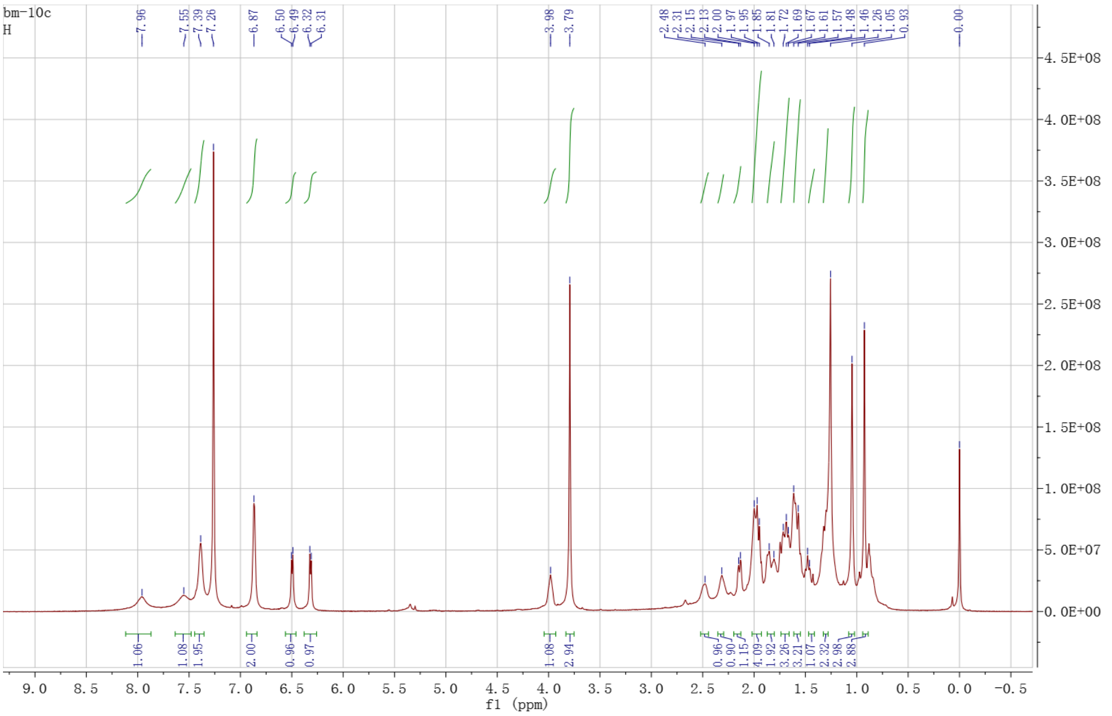

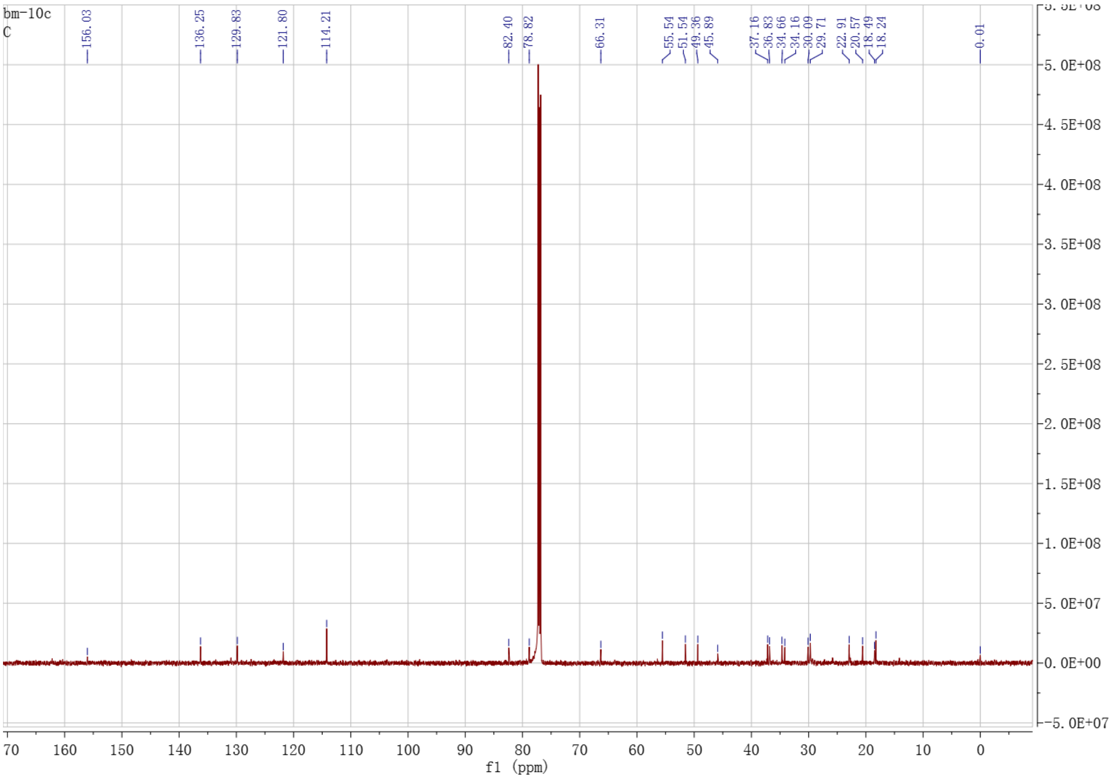

Compound 7d

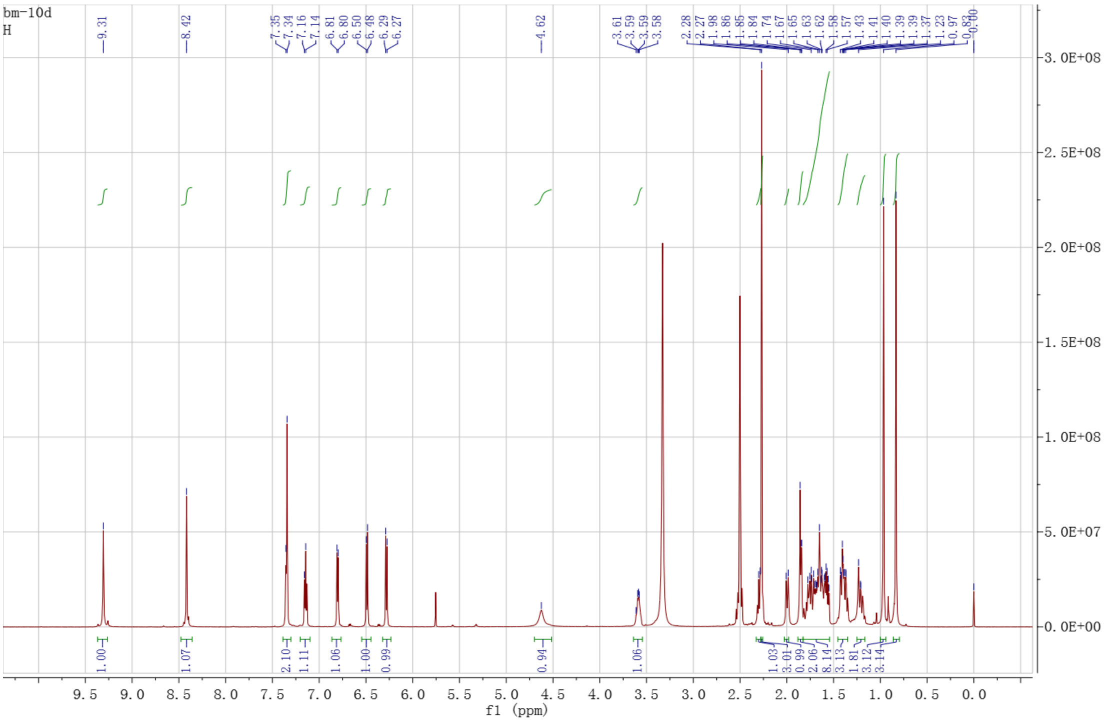

bm-10d
H
9.31
8.42
7.35
7.34
7.16
7.14
6.81
6.80
6.50
6.48
6.29
6.27
4.62
3.61
3.59
3.59
3.58
2.28
2.27
1.98
1.86
1.85
1.84
1.74
1.67
1.65
1.63
1.62
1.58
1.57
1.43
1.41
1.40
1.39
1.37
1.23
0.97
0.83
0.00
1.00
1.07
2.10
1.11
1.06
1.00
0.99
0.94
1.06
1.03
3.01
0.99
2.06
8.14
3.13
1.81
3.12
3.14
f1 (ppm)

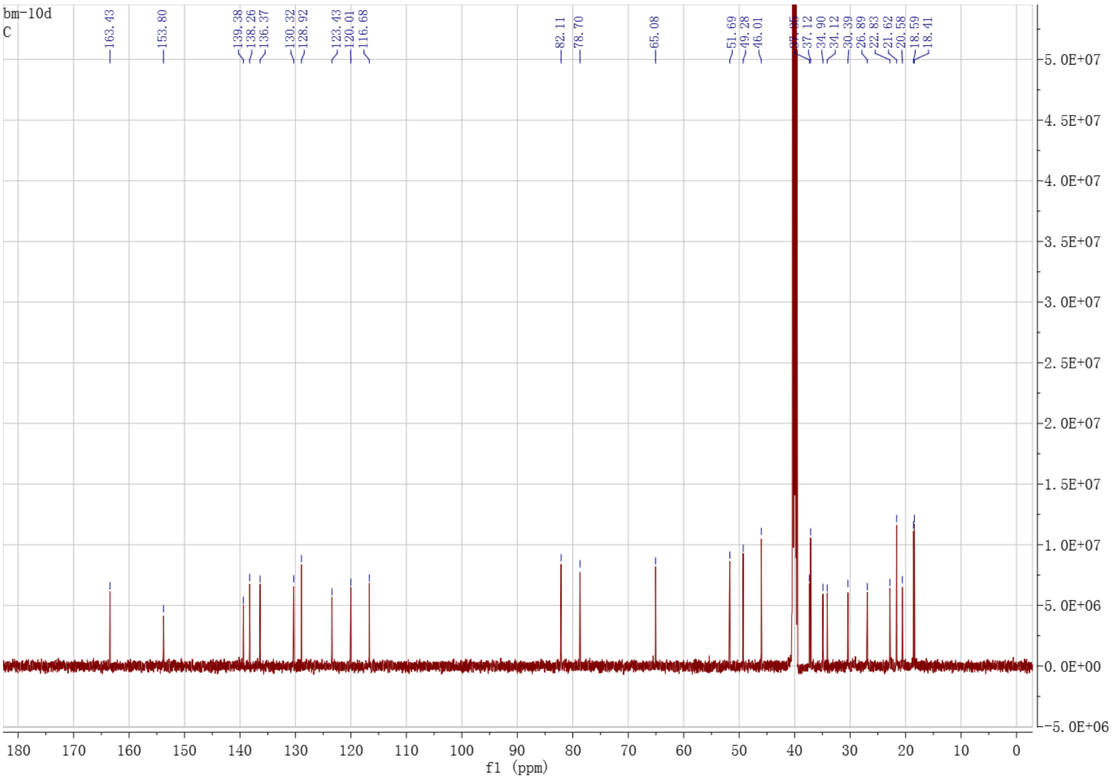

bm-10d
C
163.43
153.80
139.38
138.26
136.37
130.32
128.92
123.43
120.01
116.68
82.11
78.70
65.08
51.69
49.28
46.01
37.12
34.90
34.12
30.39
26.89
22.83
21.62
20.58
18.59
18.41
f1 (ppm)

Compound 7e

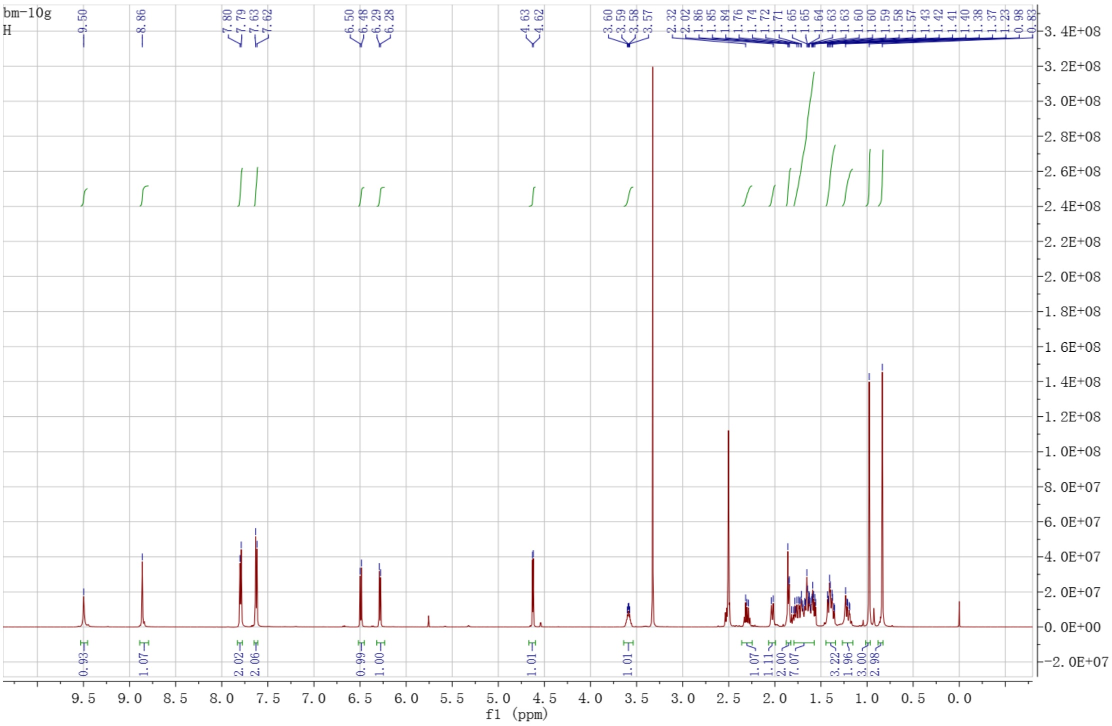

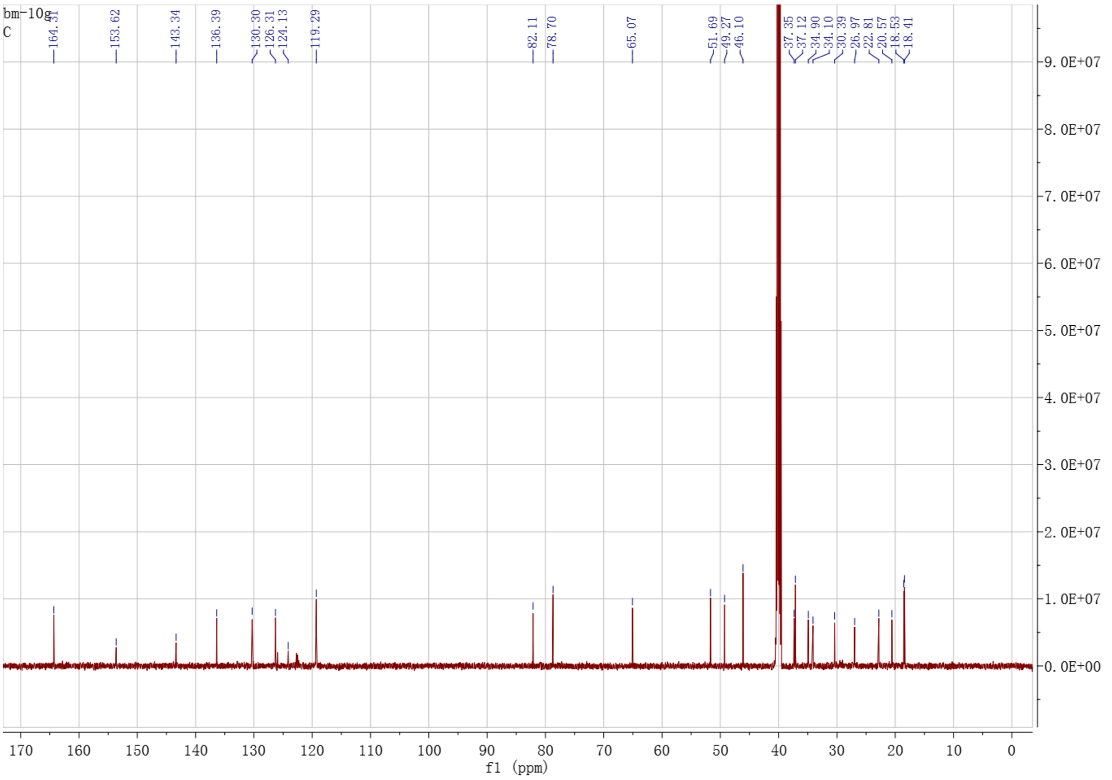

Compound 7f

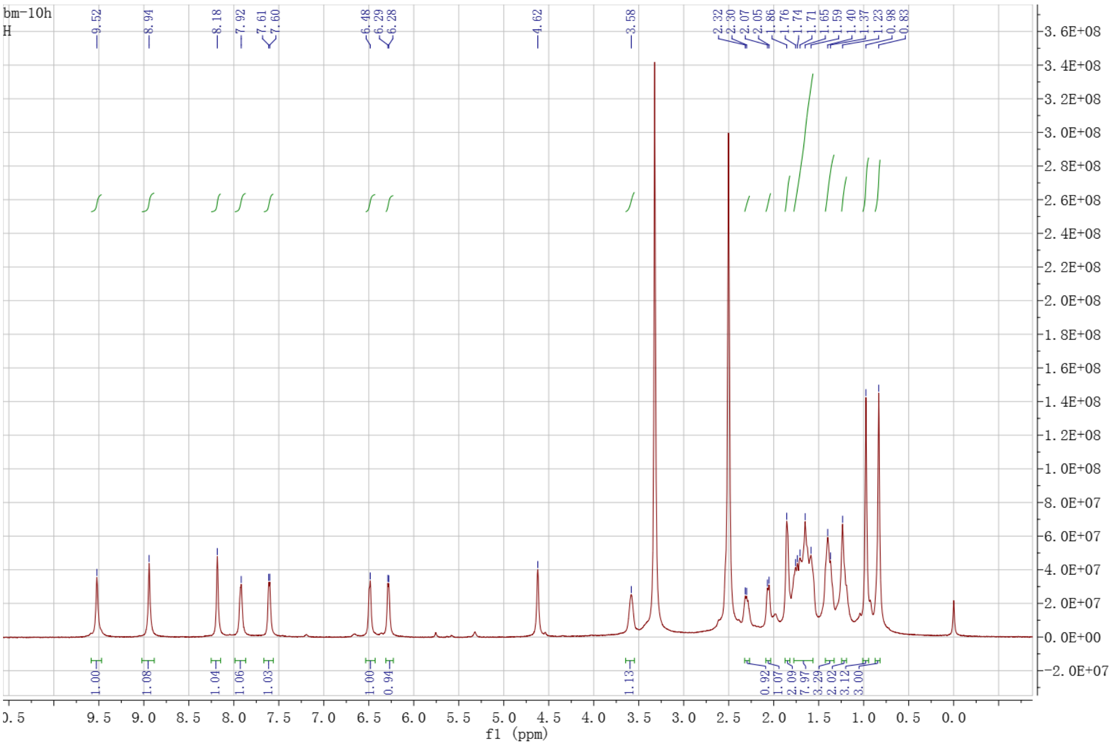
bm-10h
H
9.52
8.94
8.18
7.92
7.61
7.60
6.48
6.29
6.28
4.62
3.58
2.32
2.30
2.07
2.05
1.86
1.76
1.74
1.71
1.65
1.59
1.40
1.37
1.23
0.98
0.83
1.00
1.08
1.04
1.06
1.03
1.00
0.94
1.13
0.92
1.07
2.09
7.97
3.29
2.02
3.12
3.00
f1 (ppm)

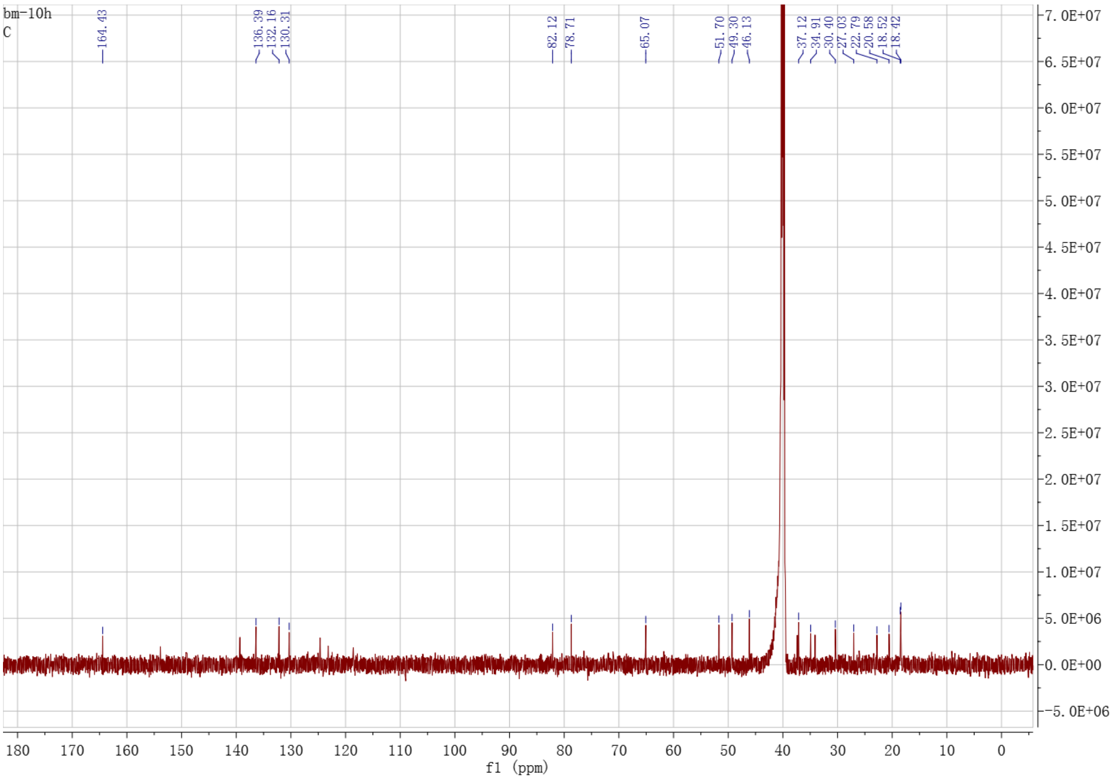
bm-10h
C
164.43
136.39
132.16
130.31
82.12
78.71
65.07
51.70
49.30
46.13
37.12
34.91
30.40
27.03
22.79
20.58
18.52
18.42
f1 (ppm)

Compound 7g

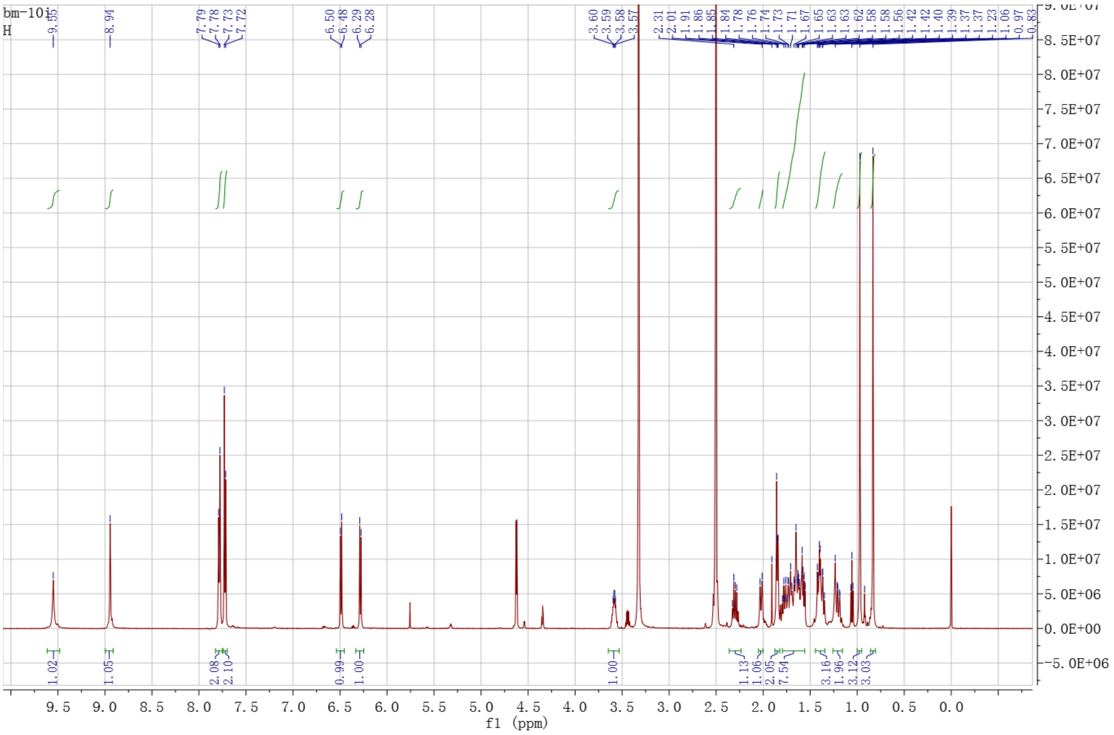

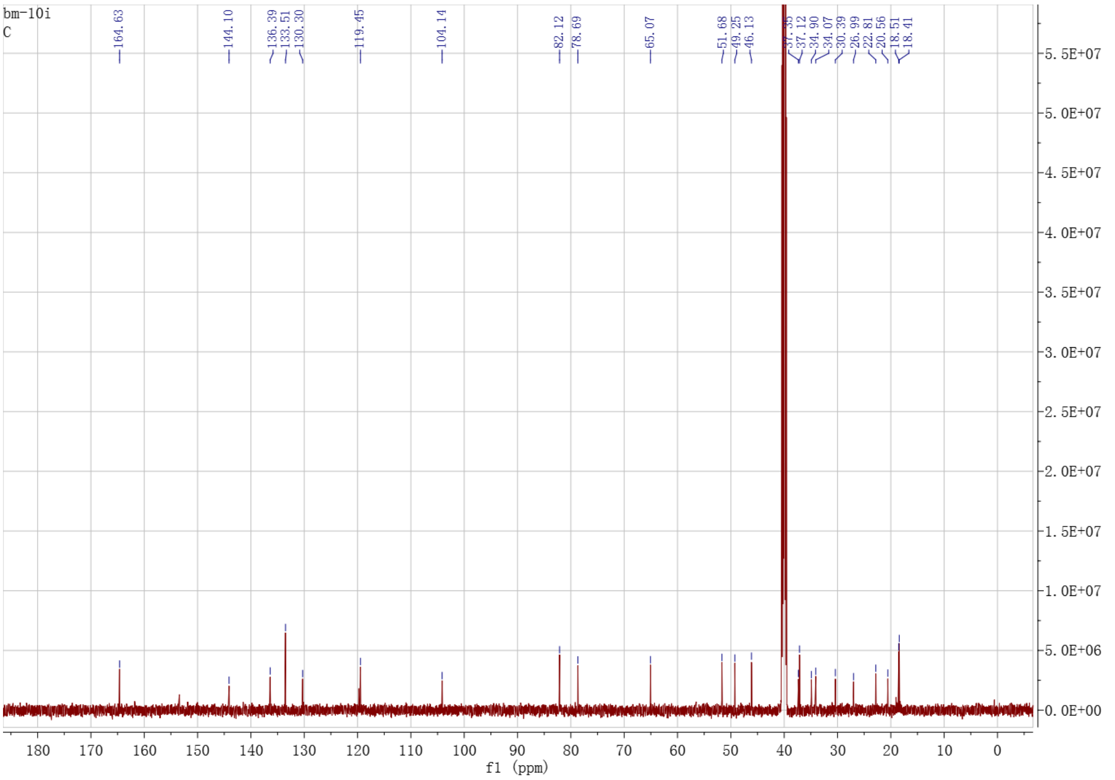

Compound 7h

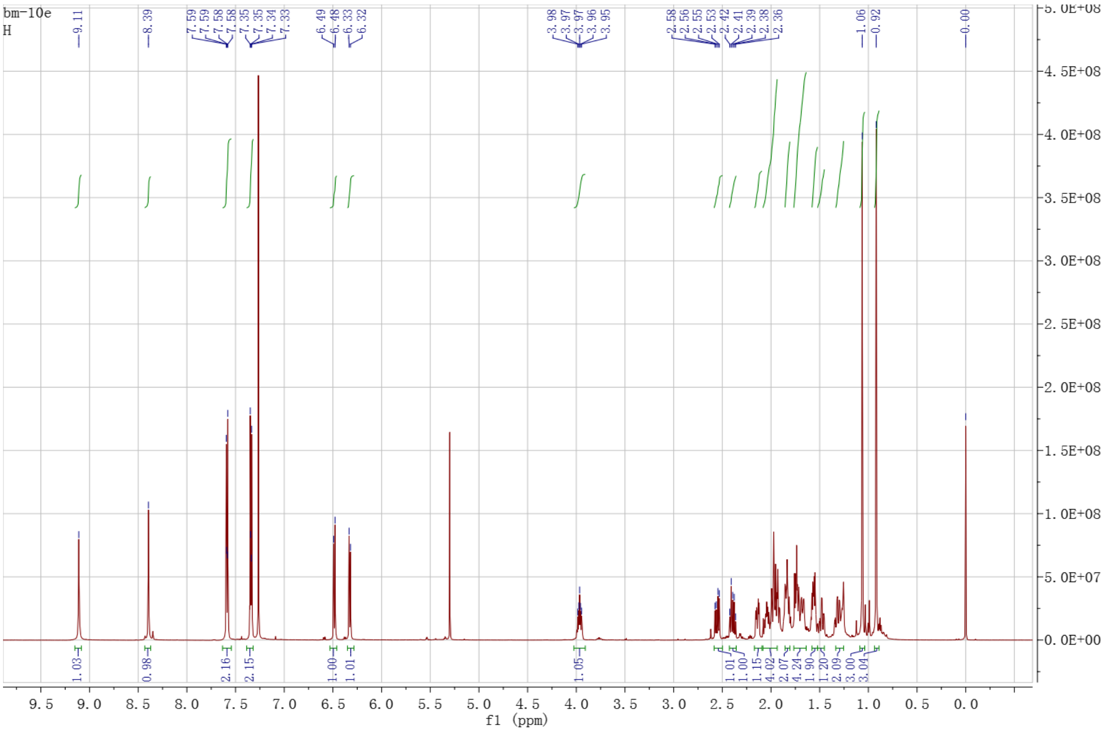

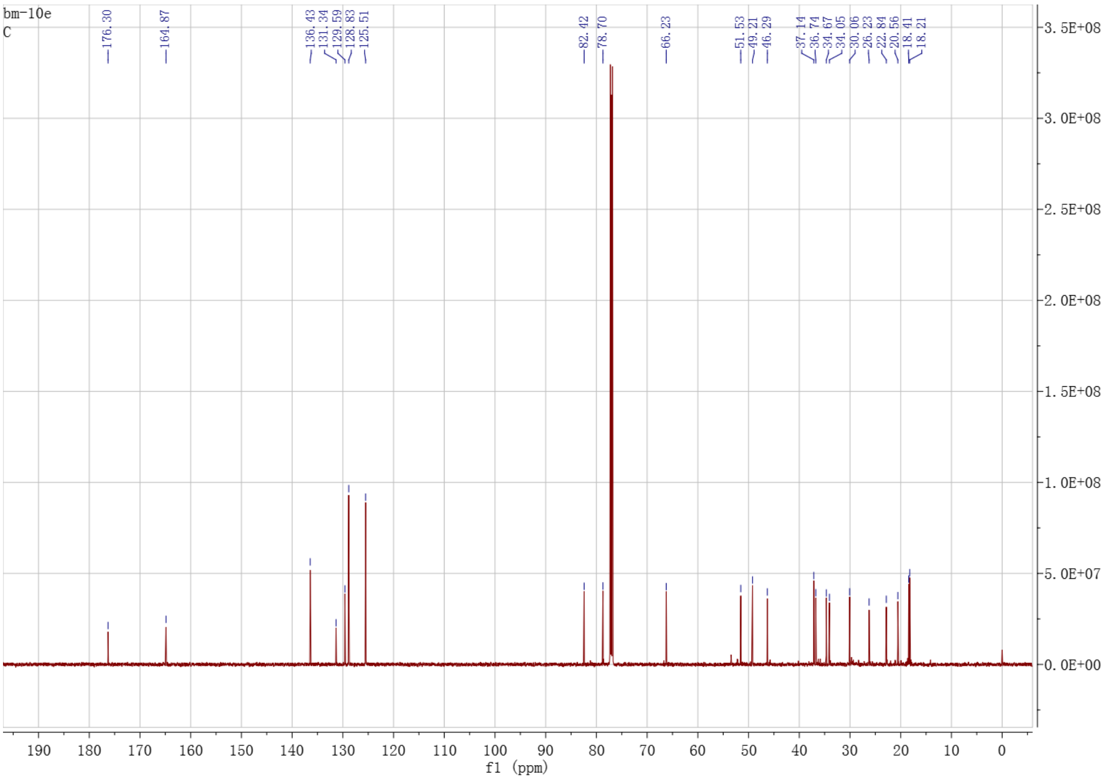

Compound 7i

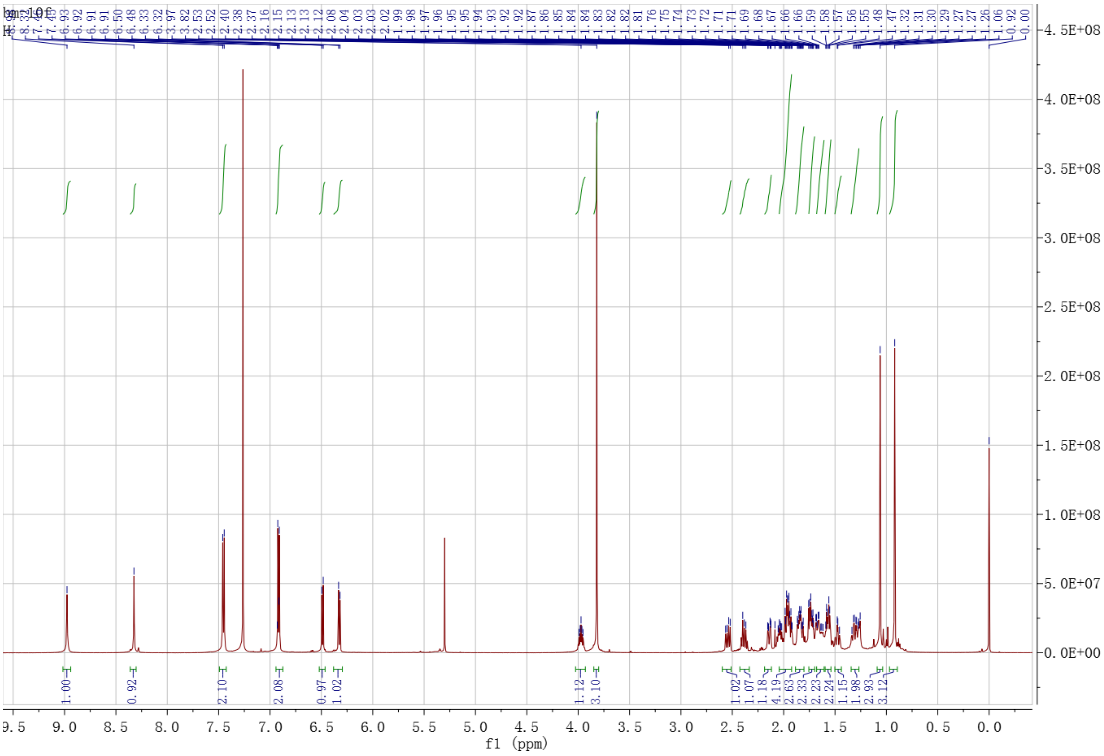

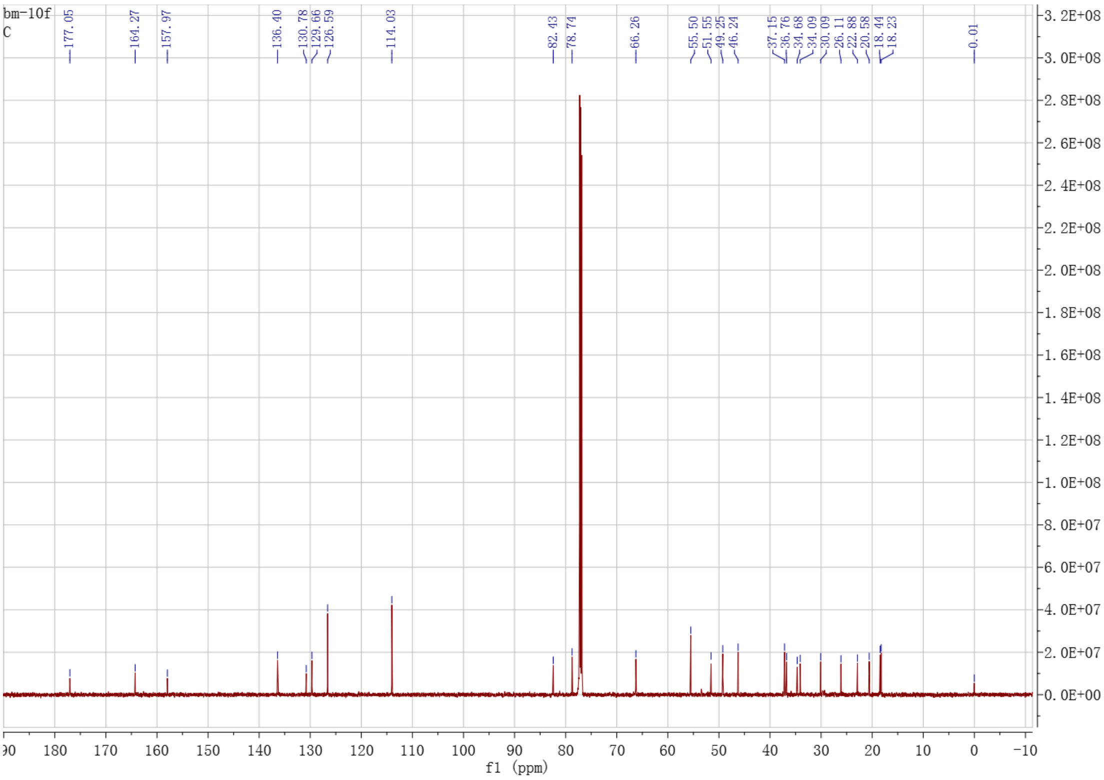

Compound 7j

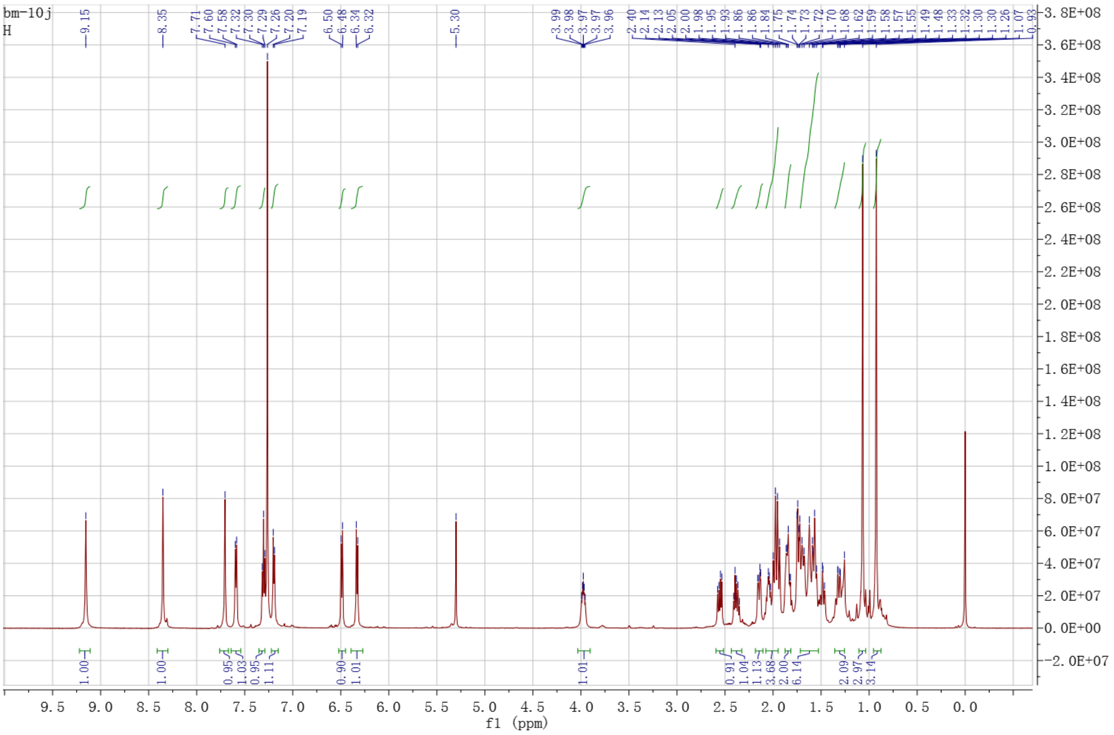
bm-10j
H
9.15
8.35
7.71
7.60
7.58
7.32
7.30
7.29
7.26
7.20
7.19
6.50
6.48
6.34
6.32
5.30
3.99
3.98
3.97
3.96
2.40
2.14
2.13
2.05
2.00
1.98
1.95
1.93
1.86
1.86
1.84
1.75
1.74
1.73
1.72
1.70
1.68
1.62
1.59
1.58
1.57
1.55
1.49
1.48
1.33
1.32
1.30
1.30
1.26
1.07
0.93
1.00
1.00
0.95
1.03
0.95
1.11
0.90
1.01
1.01
0.91
1.04
1.13
3.68
2.00
6.14
2.09
2.97
3.14
f1 (ppm)

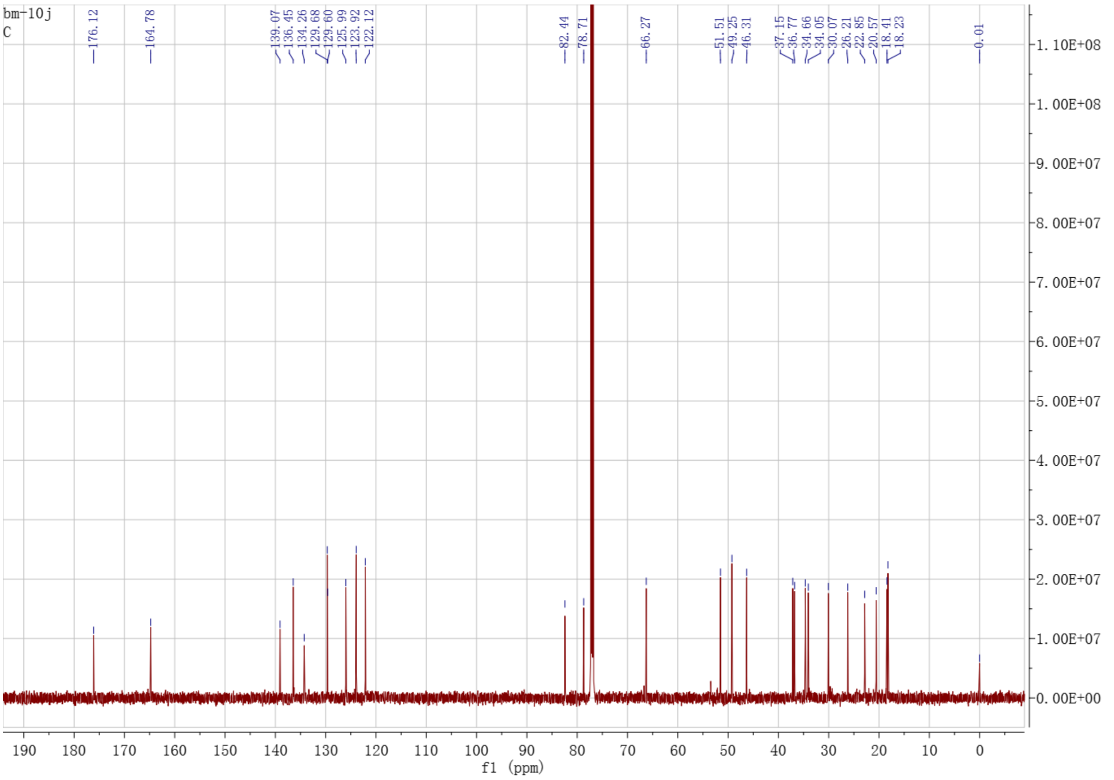
bm-10j
C
176.12
164.78
139.07
136.45
134.26
129.68
129.60
125.99
123.92
122.12
82.44
78.71
66.27
51.51
49.25
46.31
37.15
36.77
34.66
34.05
30.07
26.21
22.85
20.57
18.41
18.23
-0.01
f1 (ppm)

Compound 7k

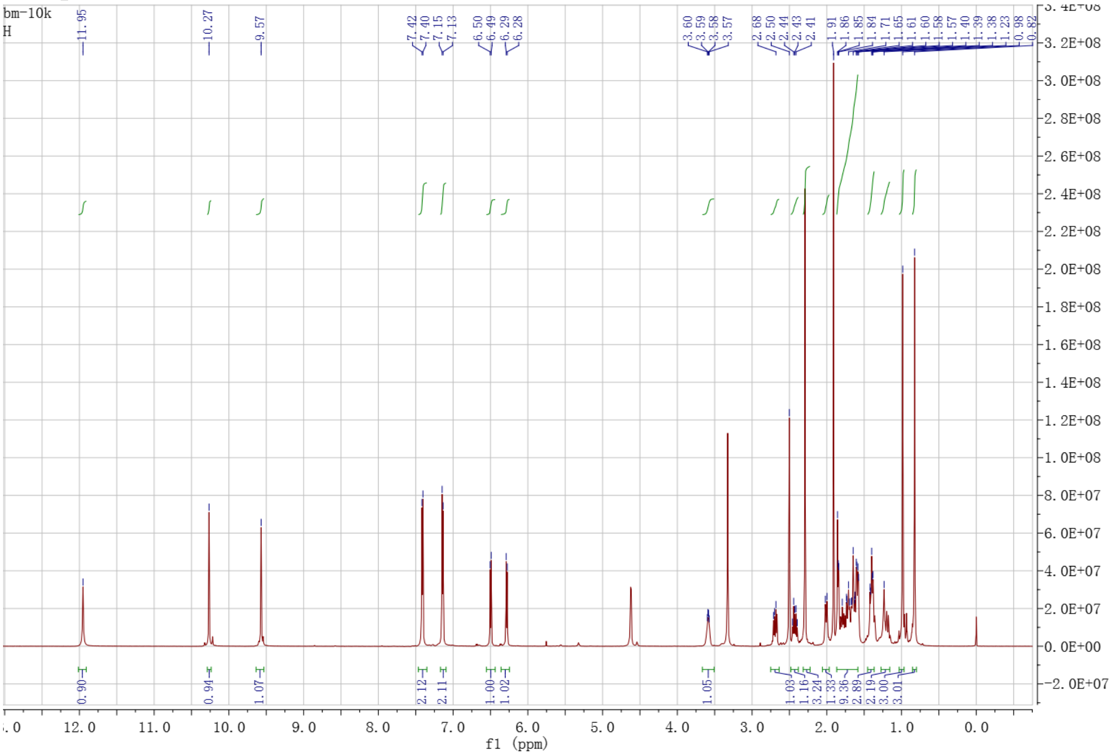
bm-10k
H
11.95
10.27
9.57
7.42
7.40
7.15
7.13
6.50
6.49
6.29
6.28
3.60
3.59
3.58
3.57
2.68
2.50
2.44
2.43
2.41
1.91
1.86
1.85
1.84
1.71
1.65
1.61
1.60
1.58
1.57
1.40
1.39
1.38
1.23
0.98
0.82
0.90
0.94
1.07
2.12
2.11
1.00
1.02
1.05
1.03
1.16
3.24
3.33
9.36
2.89
2.19
3.00
3.01
f1 (ppm)

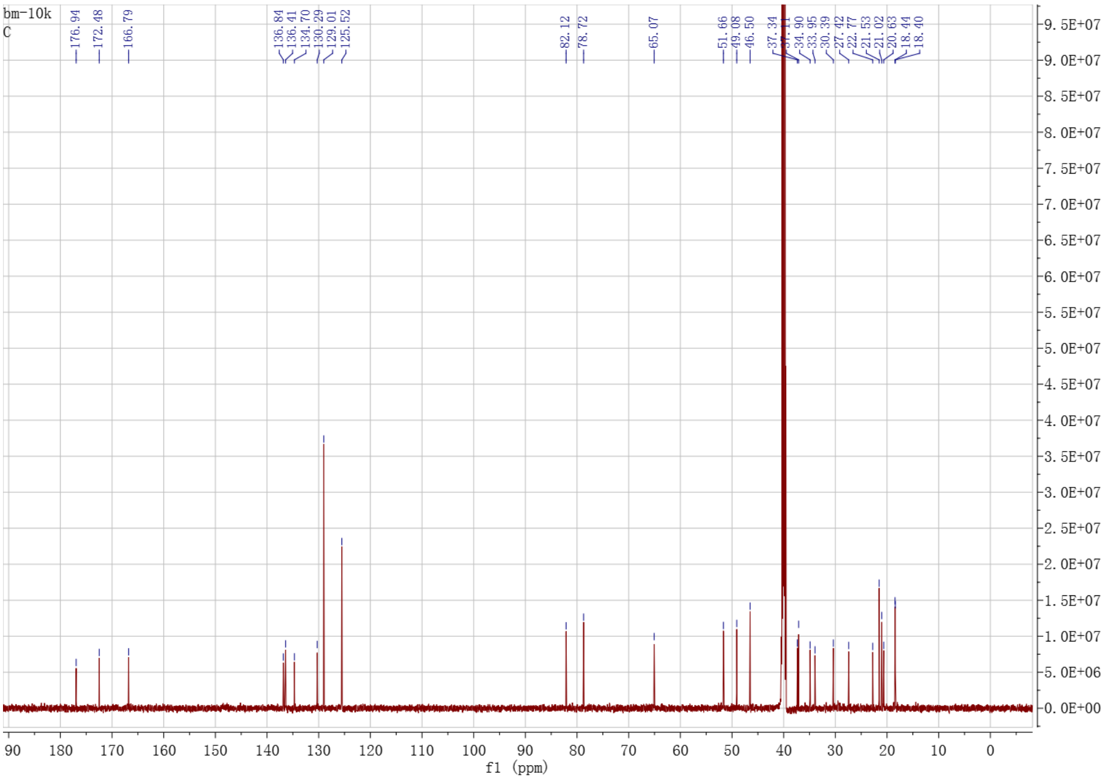
bm-10k
C
176.94
172.48
166.79
136.84
136.41
134.70
130.29
129.01
125.52
82.12
78.72
65.07
51.66
49.08
46.50
37.34
34.90
33.95
30.39
27.42
22.77
21.53
21.02
20.63
18.44
18.40
f1 (ppm)
